# Electrostatically Accelerated Encounter and Folding for Facile Recognition of Intrinsically Disordered Proteins

**DOI:** 10.1371/journal.pcbi.1003363

**Published:** 2013-11-21

**Authors:** Debabani Ganguly, Weihong Zhang, Jianhan Chen

**Affiliations:** Department of Biochemistry and Molecular Biophysics, Kansas State University, Manhattan, Kansas, United States of America; Max Planck Institute for Biophysical Chemistry, Germany

## Abstract

Achieving facile specific recognition is essential for intrinsically disordered proteins (IDPs) that are involved in cellular signaling and regulation. Consideration of the physical time scales of protein folding and diffusion-limited protein-protein encounter has suggested that the frequent requirement of protein folding for specific IDP recognition could lead to kinetic bottlenecks. How IDPs overcome such potential kinetic bottlenecks to viably function in signaling and regulation in general is poorly understood. Our recent computational and experimental study of cell-cycle regulator p27 (Ganguly *et al.*, J. Mol. Biol. (2012)) demonstrated that long-range electrostatic forces exerted on enriched charges of IDPs could accelerate protein-protein encounter via “electrostatic steering” and at the same time promote “folding-competent” encounter topologies to enhance the efficiency of IDP folding upon encounter. Here, we further investigated the coupled binding and folding mechanisms and the roles of electrostatic forces in the formation of three IDP complexes with more complex folded topologies. The surface electrostatic potentials of these complexes lack prominent features like those observed for the p27/Cdk2/cyclin A complex to directly suggest the ability of electrostatic forces to facilitate folding upon encounter. Nonetheless, similar electrostatically accelerated encounter and folding mechanisms were consistently predicted for all three complexes using topology-based coarse-grained simulations. Together with our previous analysis of charge distributions in known IDP complexes, our results support a prevalent role of electrostatic interactions in promoting efficient coupled binding and folding for facile specific recognition. These results also suggest that there is likely a co-evolution of IDP folded topology, charge characteristics, and coupled binding and folding mechanisms, driven at least partially by the need to achieve fast association kinetics for cellular signaling and regulation.

## Author Summary

Intrinsically disordered proteins (IDPs) are key components of regulatory networks that dictate various aspects of cellular decision-making. They are over-represented in major disease pathways, and are considered novel albeit currently difficult drug targets. Recognition of IDPs has extended the traditional protein structure-function paradigm, and various concepts have been proposed on how intrinsic disorder may confer crucial functional advantages. However, the physical basis of these concepts remains poorly established. In particular, while IDPs alone exist as ensembles of fluctuating structures, they frequently fold upon specific binding. Analysis of the physical timescales of protein folding and protein-protein encounter predicts that the requirement of peptide folding for specific binding could lead to a major kinetic bottleneck. In this work, carefully calibrated topology-based coarse-grained models were applied to directly simulate reversible folding and binding and investigate the recognition mechanisms of three IDP complexes. The results strongly support an electrostatically accelerated encounter and folding mechanism, where long-range electrostatic forces not only accelerate protein-protein encounter via “electrostatic steering” but also promote “folding-competent” encounter topologies to enhance the efficiency of IDP folding upon encounter.

## Introduction

Cellular signaling and regulation are frequently mediated by proteins that, in part or as a whole, lack stable structures under physiological conditions [1]–[3]. Such intrinsically disordered proteins (IDPs) are highly prevalent in proteomes [4] and over-represented in diseases pathways [5], [6]. For example, nearly one-third of eukaryotic proteins have been predicted to contain extended disordered regions [7], and about 25% of disease-associated missense mutations can be mapped into predicted disordered regions [8] (although cancer mutations appear to prefer ordered regions [9]). The prevalence of intrinsic disorder suggests that protein conformational heterogeneity could provide crucial functional advantages, for which many concepts have been proposed [10]–[14]. Understanding the physical basis of how intrinsic disorder mediates protein function (and how such functional mechanism may fail in human diseases [15]) is of fundamental significance and has attracted intense interests in recent years [16]. Important progresses have been made on characterizing the conformational properties of unbound IDPs and determining how these conformational properties contribute to efficient and reliable interactions [16]–[22].

A key recent recognition is that frequent requirement of protein folding for specific recognition of IDPs could lead to kinetic bottlenecks [23]–[25]. As predicted by the dual-transition-state theory [23], the diffusion-limited encounter rate constant represents the upper bound for that of a coupled binding and folding interaction. Importantly, the upper bound can be achieved only if the IDP readily folds upon encounter, which requires folding rates on the order of 10 µs^−1^ or greater [23]. That is, IDPs need to achieve folding rates beyond the typical µs^−1^ “speed limit” estimated for folding of isolated proteins [26] to maximize association kinetics. Therefore, the putative functional advantages of intrinsic disorder, especially structural plasticity for specific interactions with numerous partners [27], come with a potential cost of slow binding kinetics. Such kinetic bottleneck must be resolved for IDPs to be viable in cellular signaling and regulation. Interestingly, a recent survey of binding kinetic data revealed that IDP binding was not systematically slower than that of globular proteins [28]. The implication is that most IDPs do manage to fold rapidly upon nonspecific binding, and this is apparently consistent with the accumulating observations that IDP coupled binding and folding tends to follow induced folding-like baseline mechanisms (*i.e.*, bind then fold) [16], [19]. Several factors could contribute to efficient folding of IDPs upon binding, in particular small interacting (and folding) domains and simple folded topologies with low contact orders. There also appears to be a delicate balance between pre-folding and conformational flexibility that allows an IDP to quickly fluctuate among accessible conformational states, especially upon encounter [16], [29], [30]. Nonetheless, it is not yet clear how in general IDPs may achieve fast folding at rates beyond the traditional µs^−1^ folding “speed limit” upon encountering their specific targets.

An important characteristics of IDPs is that they are enriched with charged and polar residues [31]. Electrostatics can thus be expected to play key roles in IDP structure and function. For example, the charge content can modulate compaction and other conformational properties of free IDPs [32], [33]; DNA search efficiency is controlled by charge composition and distribution in disordered tails of DNA-binding proteins [34], [35]. It has been also observed or speculated in a few cases that electrostatics might be important for fast IDP recognition [36]–[39]. However, these discussions have been often based on the classic electrostatic steering effects [40], and the actual underlying mechanisms of putative electrostatic acceleration were not known. Our recent computational and experimental study of the p27-Cdk2/cyclin A interaction revealed that long-range electrostatic forces could promote facile IDP recognition via an “electrostatically accelerated encounter and folding mechanism” [24]. Specifically, the measured p27/Cdk2/cyclin A association rate constants showed a strong salt-dependence, increased ∼12 fold when the ionic strength was reduced from 0.6 to 0.075 M. However, the salt-dependence is poorly described by an approximate Debye-Hückel relation [41] that mainly captures the electrostatic steering effects. Instead, simulations using a series of topology-based coarse-grained models suggested that long-range electrostatic forces exerted on a large number of charges on p27 did not only accelerate the encounter rate (via the classical electrostatic steering effect [40]), but enhance the efficiency of p27 folding upon encounter by promoting native-like encounter topologies.

Analysis of surface charges in a set of existing IDP complexes further revealed that the vicinity of IDP binding sites tended to be enriched with charges to complement those on IDPs [24] (even though the IDP binding interface itself is more hydrophobic than the rest of the protein surface as previously observed [42]). Electrostatic forces are known to be a dominant long-range force that can guide protein orientation in protein-DNA interactions [43], [44] and/or modulate early stages of protein folding [45]–[47]. One implication of enriched charges near IDP binding sites is thus that the electrostatically accelerated encounter and folding mechanism observed for p27 may be prevalent in signaling and regulatory IDPs. Nonetheless, the ability for long-range electrostatic forces to enhance folding upon binding can be surprising, as nonspecific interactions (electrostatic or van der Waals) have been generally expected to accelerate binding but slow down folding [48], [49]. It has also been predicted that, while inter-chain electrostatic interactions facilitate binding of disordered chaperone Chz1 to histone variant H2A.Z-H2B, intra-chain electrostatic interactions could lead to premature collapse of Chz1 under low salt conditions and hinder the overall rate of forming the specific complex [50].

**Figure 1:**
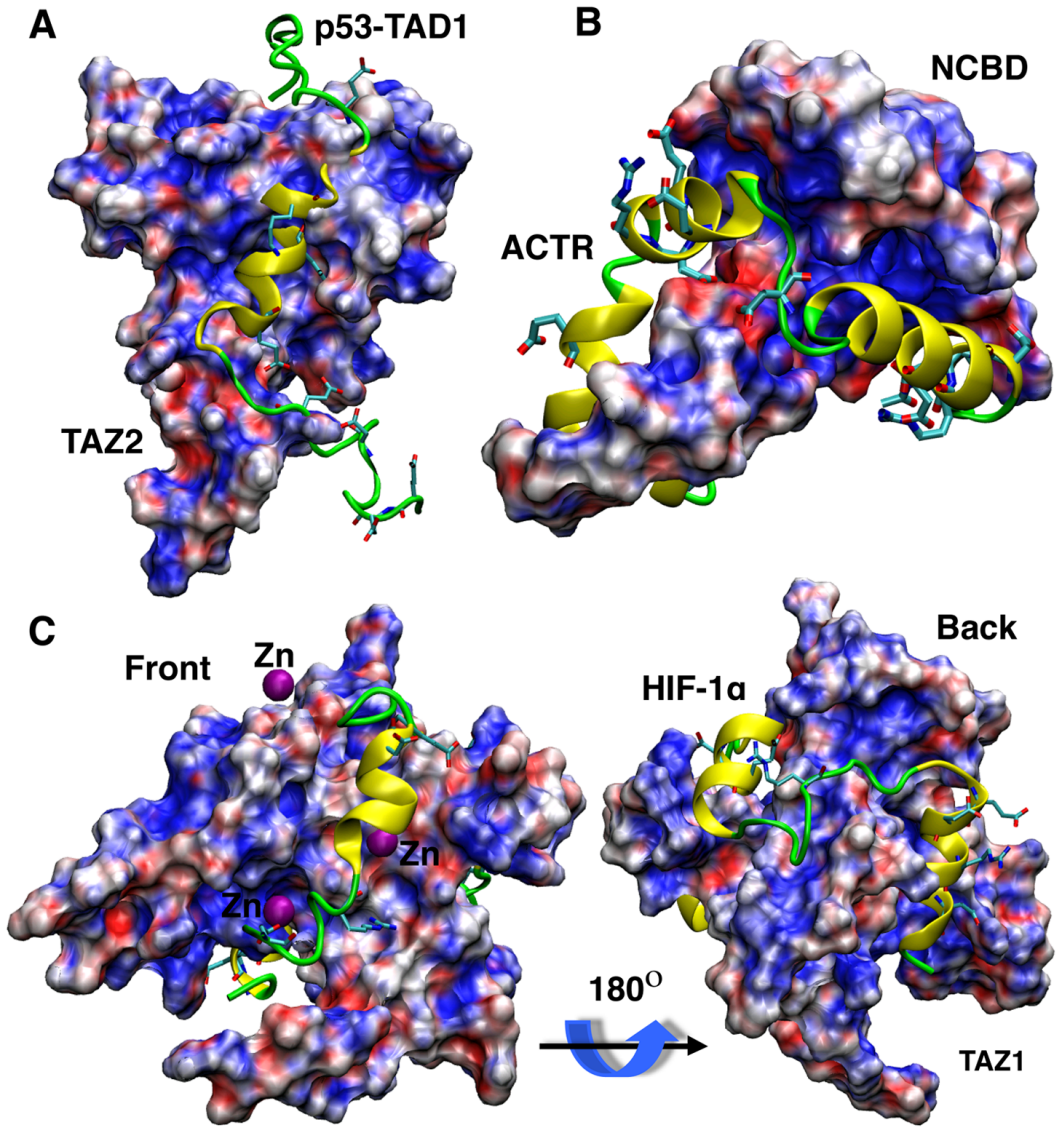
Structures and surface electrostatic potentials of three complexes. A) p53-TAD1/TAZ2, B) NCBD/ACTR, and C) HIF-1α/TAZ1. TAZ2, NCBD and TAZ1 are shown in molecular surface and colored based on the surface electrostatic potential calculated using PBEQ module of CHARMM [80], [81]. Red indicates negative and blue indicate positive charge. p53-TAD1, ACTR and HIF-1α are shown in cartoons, with charged side chains shown in stick.

In the present work, we investigated the recognition mechanisms and the roles of long-range electrostatic interactions in forming of three IDP complexes, namely, p53-TAD1/TAZ2, HIF-1α/TAZ1, and NCBD/ACTR (Table 1). All these complexes have important biological functions. For example, tumor suppressor p53 is considered one of the most important proteins in cancer [51]; NCBD and TAZ1/2 are key regulatory domains of CBP, a key component of the general transcriptional machinery that plays critical roles in cell fate regulation [52]. For understanding IDP recognition, these systems involve more complex folded topologies than that of p27 in the p27/Cdk2/cyclin A complex. As shown in Fig. 1, both HIF-1α/TAZ1 and NCBD/ACTR possess extensive binding interfaces, whereas the binding interface in p53-TAD1/TAZ2 is more localized. Importantly, while strong charge complementary exists near the binding interface (as expected), the surface electrostatic potentials of the folded substrates do not show prominent features like those observed on Cdk2/cyclin A (e.g., see Fig. 1 of reference [24]) to directly suggest that long-range electrostatic forces could promote native-like (and thus more folding-competent) encounter complexes. The NCBD/ACTR complex involves synergistic folding of two IDPs and thus offers a particularly interesting opportunity to understand whether and how electrostatic interactions may modulate the formation of nontrivial folded topologies. Amazingly, all three complexes associate with on-rates in excess of 10^7^ M^−1^s^−1^ (see Table 1), a regime that is typically considered “diffusion-limited” and can only be accessed in the limit of ultrafast conformational transitions [40].

**Table 1:** Key properties of three IDP complexes.

Name^a^	Length	*K*_D_ b	*k*_on_ (M^−1^s^−1^)	PDB	IDP Fold	Charges^c^
**p53-TAD1**/TAZ2	39/90	2.7 µM [77]	∼10^8^ d	2k8f	helix/loops	8(−6), 9(+9), 4(+2)
**HIF-1α**/TAZ1	51/99	7 nM [70]	1.3×10^9^ [78]	1l8c	helices/loops	11(−5), 11(+7), 10 (+5)
**NCBD**/**ACTR**	59/47	34 nM [79]	3×10^7^ [65]	1kbh	helices	- (both IDPs)

a Abbreviations: ACTR: the activation domain of p160 steroid receptor co-activator; HIF-1α: hypoxia-inducible factor 1 α subunit; NCBD: the nuclear-receptor co-activator binding domain of CREB binding protein (CBP); p53-TAD1: the transactivation domain 1 of tumor suppressor p53; TAZ1/2: the TAZ domains of CBP. The sequences of all IDPs involved (highlighted in bond fonts) are provided in the Supporting Information. Text S1.

b The experimental *K*_D_ values were measured at 308 K for p53-TAD1/TAZ2, 298 K for HIF-1α/TAZ1, and 304 K for NCBD/ACTR. Note that *K*_D_ only weakly depends on temperature for p53-TAD1/TAZ2 (doubled when the temperature is increased from 288K to 308K [77]).

c Numbers of charged residues and the net charges (in parentheses) of the IDP, its binding site, and the vicinity of the binding site. Residues at the IDP binding interface are identified as those with greater than 1.0 Å^2^ solvent accessible surface area changes upon complex formation. Surface residues are identified as those with >5% solvent accessibility. All surface residues within 15 Å Cα-Cα distance from the bound IDP but not directly involved in intermolecular contacts are considered to be within the vicinity of the IDP binding site.

d Estimated based on the association rate constant of p53-TAD2/TAZ2 (∼10^10^ M^−1^s^−1^ [38]), assuming that TAD1 and TAD2 have similar off rates. TAD2 binds to the TAZ2 primary site with *K*_D_ ∼32 nM [38], about two orders of magnitude stronger than TAD1.

## Results

### Topology-based modeling of IDP coupled binding and folding

Series of topology-based coarse-grained models were first derived based on the complex structures to allow direct simulation of reversible binding and folding with tractable computational cost. Topology-based modeling is based on the theoretical framework of minimally frustrated energy landscapes for natural proteins [53], and has been highly successful in predicting essential features of protein folding mechanisms [53]–[55]. Formation of stable IDP complexes such as those studied in this work should also satisfy minimal frustration, and thus topology-based modeling is applicable. Indeed, it has been successfully applied to several IDP complexes [56]–[60], with many key predictions substantiated by independent experimental studies. Nonetheless, important differences do exist between IDPs and structured proteins in sequence compositions and binding interface characteristics [42]. We have previously demonstrated that traditional topology-based models need to be carefully calibrated to ensure proper balance among competing intramolecular and intermolecular interactions (see Methods for detail on the calibration protocol) [61]. We note that the importance of model calibration was also illustrated in a recent study of the HIF-1α/TAZ1 complex [59].

Table 2 summarizes the final calibrated models for all three complexes. The calculated residual helicity distributions of the unbound states are show in Fig. S1. Three independent models were constructed for each complex: one without explicit charges (mimicking high salt concentration with fully screened long-range electrostatic interactions), one with explicit charges (mimicking low salt concentration with unscreened long-range electrostatic interactions), and a third one with explicit charges and 0.05 M salt (mimicking physiological conditions). All models reproduce the experimental *K*_D_ to the same order of magnitude, except that the no charge model for HIF-1α/TAZ1 yields a *K*_D_ value about one order of magnitude too large. We note that calculated *K*_D_ values can be very sensitive to small changes of in the scaling of intermolecular interactions during model calibration (see Methods). It is computationally expensive to use REX simulations to systematically search for the parameter space, especially for models without explicit charges due to slower transitions. Nonetheless, by performing production simulations at the corresponding melting temperatures, remaining imperfections in the balance of various interactions should be further suppressed, allowing reliable comparative studies of the mechanistic roles of electrostatic interactions in coupled binding and folding.

**Table 2:** Dissociation constants, melting temperatures, average reversible coupled binding and folding transition rates calculated using various coarse-grained models with and without explicit charges and/or 0.05 M salt.

Models	Calc. *K*_D_	*T*_m_ (K)	*k*_TS_ (µs^−1^)	*k*_cap_ (ns^−1^)	*k*_esc_ (ns^−1^)	*k*_evo_ (ns^−1^)
**TAD1/TAZ2**						
No charge	1.4±2.0 µM	327	4.3±1.5	1.4	8.0	0.049
Charged, 0.05M salt	1.6±1.6 µM	340	14.5±1.1	3.2	4.1	0.08
Explicit charges	4.9±3.2 µM	335	27.0±0.2	32.1	0.10	0.16
**HIF-1α/TAZ1**						
No charge	64±64 nM	327	6.1±0.5	2.8	6.3	0.022
Charged, 0.05M salt	9.4±9.6 nM	340	10.2±1.8	3.4	5.6	0.039
Explicit charges	1.3±1.6 nM	345	29.4±3.7	5.0	0.69	0.048
**NCBD/ACTR**						
No charge	67±99 nM	318	0.53±0.2	0.13	0.61	0.0043
Charged, 0.05M salt	96±92 nM	315	1.7±0.1	0.29	0.31	0.0074
Explicit charges	39±14 nM	322	5.2±0.7	0.79	0.020	0.012

*K*_D_ was calculated from REX simulations at 300 K(see Table 1 for the experimental values); *k*_TS_ was calculated from the production Langevin simulations at the corresponding *T*_m_, as *k*_TS_ = *N*_TS_/*t*_tot_, where *N*_TS_ is the number of reversible binding and folding transitions observed during the total simulation time span *t*_tot_. As all simulations were performed at *T*_m_, *k*_TS_ as defined is half of the binding and unbinding rates. *k*_cap_ *k*_esc_ and *k*_evo_ are defined in Eqns. 1–4. The effective concentrations of these simulations are 1.66 mM, 1.66 mM and 1.43 mM for p53-TAD1/TAZ2, HIF-1α/TAZ1 and NCBD/ACTR, respectively. All uncertainties were estimated as the differences between results calculated from the first and second halves of the data.

### Baseline mechanisms of coupled binding and folding: Effects of electrostatic forces

**Figure 2:**
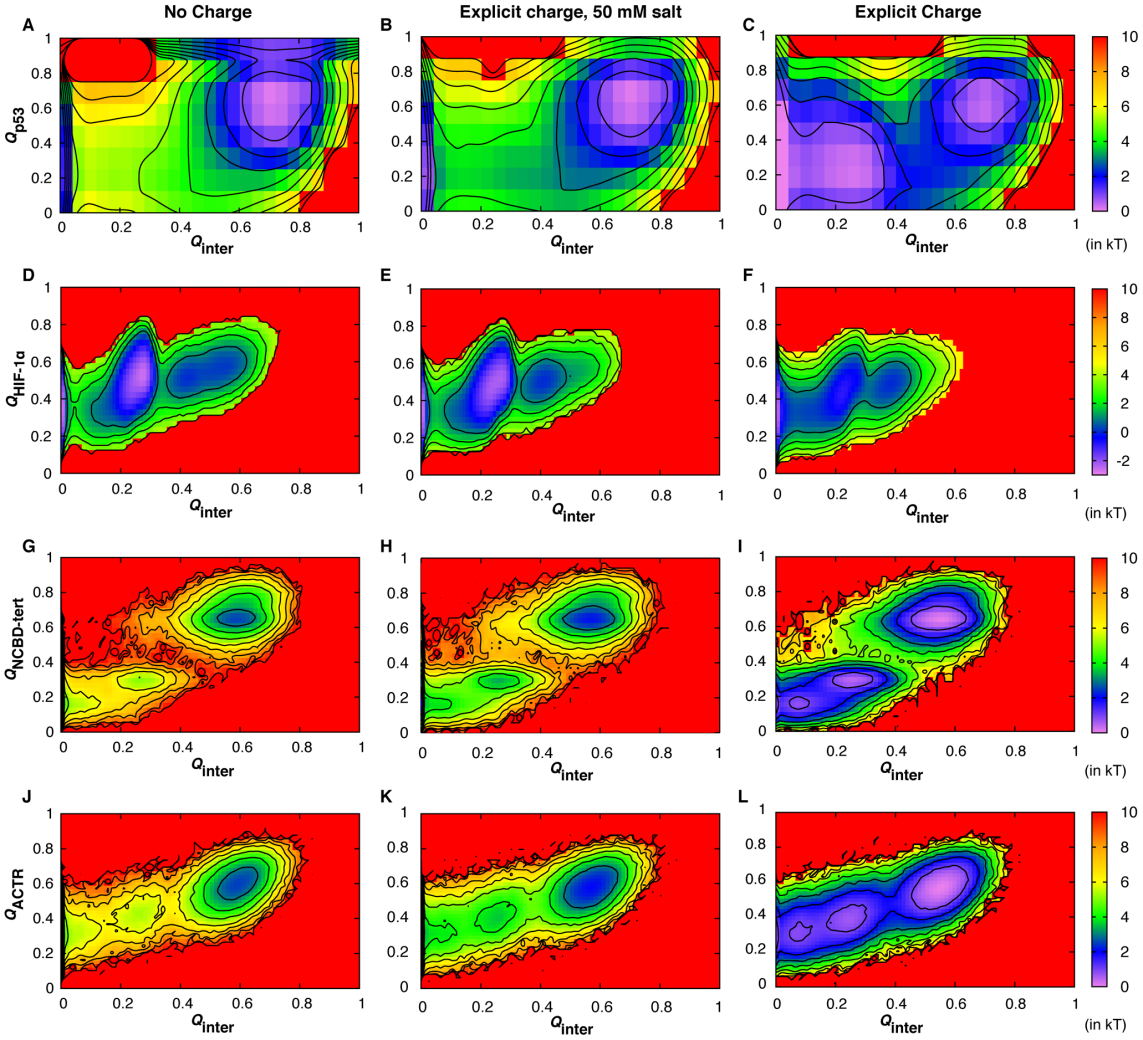
Free-energy surfaces at *T*_m_ as a function of the fractions of intra- and intermolecular contacts formed, computed using various Gō-like models with and without explicit charges and/or 50 mM salt (see Table 2). Rows A–C, D–F and G–L are for the p53-TAD1/TAZ2, HIF-1α/TAZ1 and NCBD/ACTR complexes, respectively. *Q*_inter_ is the fraction of intermolecular contacts formed; *Q*_p53_, *Q*_HIF-1α_ and *Q*_ACTR_ are the fractions of intramolecular contacts formed by p53-TAD1, HIF-1α and ACTR, respectively; *Q*_NCBD-tert_ is the fraction of tertiary intramolecular contacts formed by NCBD (the helical content of NCBD remain similar during coupled binding and folding). Contours are drawn every kT, where k is Boltzmann constant and T is the absolute temperature.

Free energy surfaces were constructed using various combinations of folding and binding order parameters to understand the baseline mechanisms of coupled binding and folding and to dissect the effects of long-range electrostatic forces. In particular, the fractions of native contacts formed have been shown to provide natural reaction coordinates for such mechanistic analysis [62]. Fig. 2 compares the free energy surfaces as a function of intra- and inter-molecular native contact factions for all three complexes, calculated using calibrated Gō-like models with and without explicit charges and/or salt (see Table 2). Both p53-TAD1 and HIF-1α recognitions follow induced folding-like mechanisms, where the peptides only gain structures after forming significant numbers of native intermolecular contacts. For example, Fig. 2A shows that p53-TAD1 does not start to fold until *Q*_inter_ reaches ∼0.5. Free NCBD is a molten globule with folded-like secondary structures [63], and its synergistic folding with ACTR has been previously shown to involve multiple stages of selection and induced folding [25], [60], reminiscent of the “extended conformational selection” mechanism [30]. Nonetheless, neither protein gains significant secondary (for ACTR) or tertiary (for NCBD) structures until over 20% of native intermolecular contacts are formed (Fig. 2G and 2J).

Interestingly, formation of all three complexes involves intermediates, even though the intermediate in p53-TAD/TAZ2 interaction only become pronounced in the presence of nonspecific electrostatic forces (see Fig. 2A vs 2C). Detailed examination of the simulation trajectories and various free energy surfaces using fractions of native contacts formed by different IDP segments (e.g., see Figs. S2, S3, S4) revealed the existence of multiple parallel pathways for forming HIF-1α/TAZ1 and NCBD/ACTR. While these mechanistic details are not the focus of the current work, they appear to be highly consistent with previous experimental and computational studies. For example, as shown in Fig. S2, both the first and third helices of HIF-1α could initiate recognition, with the pathway initiated by the third helix binding being much more prevalent. Similar observations were also made in a separate computational study [59]. Specific recognition of NCBD/ACTR appears to be primarily initiated by the C-terminal segments of these two peptides (Figs. S3, S4), which forms a key intermediate that was also suggested by an H/D exchange mass spectrometry study [64]. Kinetic data from a recent stop-flow study of the NCBD/ACTR interaction [65] are consistent with the prediction of induced folding as a baseline mechanism and have further confirmed the existence of parallel pathways and multiple folding intermediates. Representative snapshots along the dominant binding and folding pathways of p53-TAD1/TAZ2 and HIF-1α/TAZ1 are shown in Figs. S5, S6.

Explicit inclusion of charges does not significantly perturb the baseline mechanisms of coupled binding and folding. As shown in Fig. 2 and Figs. S2, S3, S4, long-range electrostatic forces do not lead to fundamental changes in any of the free energy surfaces examined. The baseline mechanisms for the formation of all three complexes remain induced folding-like. Furthermore, nonspecific electrostatic interactions do not change the relative prevalence of the parallel pathways that exist. For example, HIF-1α still initiates binding mainly through the third helix (Fig. S2); synergistic folding NCBD and ACTR is still mainly initiated through their C-terminal segments (Figs. S3, S4). The key effect of electrostatic forces appears to be substantial reductions in the free energy barriers that separate various basins. That is, even under the no salt condition, strong nonspecific electrostatic interactions do not appear to add to the ruggedness of coupled binding and folding free energy surfaces. An implication is that there exists a level of self-consistency between the charge distribution and folded topology in the bound states, despite a lack of apparent complementary between folding topologies and surface electrostatic potentials for these IDP complexes (see Fig. 1).

### Kinetic effects of long-range and nonspecific electrostatic forces

**Figure 3:**
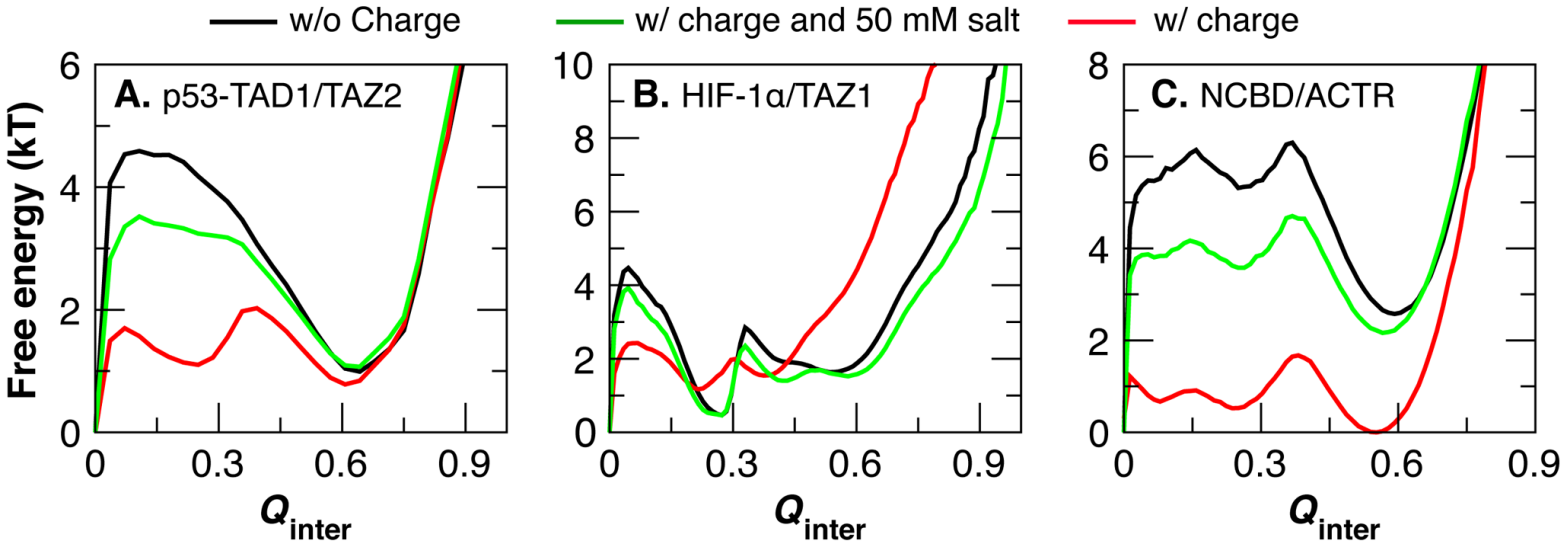
Free energy as a function intermolecular contact fraction at *T*_m_. These profiles were calculated from the REX simulations using WHAM for: A) TAD1/TAZ2, B) HIF-1α/TAZ1, and C) NCBD/ACTR.

Kinetics of coupled binding and folding was derived directly from production Langevin dynamics simulations performed using the calibrated Gō-like models at their corresponding *T*_m_. The results, summarized in Table 2, show that long-range electrostatic forces accelerate the reversible binding/unbinding transition rates for all three complexes. The overall electrostatic acceleration, estimated by comparing the average transition rates (*k*_TS_) calculated using models with and without explicit charges, ranges from ∼5 fold for HIF-1α to 10 fold for NCBD/ACTR. The magnitude of acceleration is similar to what was previously measured for other IDPs including p27 [24] and PUMA [39] (both ∼10 fold). The presence of 0.05 M salt significantly attenuates the predicted electrostatic acceleration, to only about two fold. However, the effect of salt screening on electrostatic acceleration is likely over-predicted [24], which is due to the C_α_-only model used in this work and may be corrected with more detailed protein models [45]. Consistent with the kinetic analysis, there are significant reductions in the free energy barriers along *Q*_inter_ (see Fig. 3), which has been shown to be a good binding reaction coordinate [61]. In addition, the magnitude of barrier reduction correlates well with the degree of rate acceleration calculated directly from Langevin dynamics simulations, with the largest barrier reduction observed for NCBD/ACTR and the smallest reduction observed from HIF-1α/TAZ1.

To further analyze the effects of electrostatic interactions on different stages of coupled binding and folding, the recognition process was divided into two generic steps, including an encounter step followed by an evolving (folding) step to final bound and folded state (Eq. 1 in Methods). Such generic decomposition ignores the details of IDP-specific folding pathways, to allow on to focus on the net effects of electrostatic forces on the overall efficiency of IDP folding upon encounter. For this, three general states were identified during production simulations, including the unbound (U), collision complex (CC), and bound (B) states (see Methods for specific criteria for state assignment). The mean first passage times (MFPT) and numbers of transitions (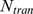) among these states were then calculated. The results, summarized in Tables S1, S2, 3, show that long-range electrostatic forces greatly reduce the average encounter time, from 0.72 to 0.03 ns for p53-TAD, from 0.37 to 0.20 ns for HIF-1α, and from 7.71 to 1.26 ns for NCBD. At the same time, long-range electrostatic forces also significantly enhance the efficiency of IDP folding upon encounter, allowing much larger fractions of the encounter complexes to eventually evolve to the bound states. For example, for NCBD/ACTR, only 16 out ∼2300 encounter events evolved to the bound state in absence of long-range electrostatic forces (0.7%); whereas with explicit charges, there was ∼37% probability (108 out of 288) of forming the specific complex once the proteins were captured into the collision complex state (Table S3). For the HIF-1α/TAZ1 complex, the percentages of collision to specific complex transition are 0.4% without and 7% with explicit charges (Table S2); for p53-TAD1/TAZ2, the production percentages are 0.6% without and 60% with explicit charges (Table S1). It should be emphasized that nonspecific electrostatic interactions significantly stabilize the collision complexes, due to large and complementary net charges of the interacting proteins (see Table 1). As such, much fewer fully unbinding events were observed during production simulations using the charged models. This effect also led to more reversible transitions between the bound and collision complex states and thus an overestimation of the true folding efficiency of IDPs upon collision as estimated above. We also note that the collision complexes as defined in our analysis were not intended to represent so-called “encounter complexes” that have been often considered key intermediates of protein-protein association [66], although encounter complexes are also believed to be mainly stabilized by nonspecific electrostatic interactions.

**Figure 4:**
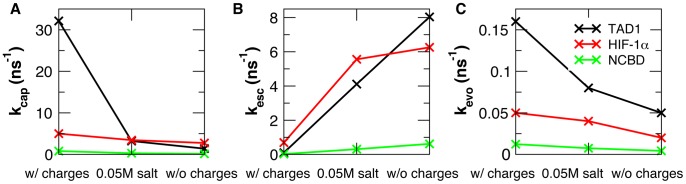
Effective rate constants for transitions between the unbound, collision complex and bound states. The rates, as defined in Methods Eqns. 1–4, were calculated using models with and without explicit charges and/or 50 mM salt for: A) TAD1/TAZ2, B) HIF-1α/TAZ1 and C) NCBD/ACTR. The results demonstrate that long-range electrostatic forces increase both the capture and evolution rates and at the same time reduce the escape rates.

The enhanced apparent efficiency of folding upon encounter appears to be frequently achieved at the cost of longer folding times. For example, the MFPTs of transitions from the collision complexes to the bound states increase from 0.26 to 3.94 ns for the p53-TAD1/TAZ2 complex (Table S1) and from 8.14 to 44.56 ns for the NCBD/ACTR complex (Tables S3). The net effects on the kinetics of encounter and folding stages can be quantified by calculating three effective rate constants as defined in Eqns. 2–4 (see Methods) [28]. The results, summarized in Table 2 and plotted in Fig. 4, clearly demonstrate that nonspecific electrostatic interaction enhance the encounter rates and reduce the escape rates of the collision complexes. Importantly, the effective evolution rates are always faster, by about three fold, in the presence of long-range electrostatic forces, despite longer MFPTs for the transitions from the collision complexes to the bound state observed for the p53-TAD1/TAZ2 and NCBD/ACTR complexes. The magnitude of electrostatic acceleration of folding upon encounter is similar to what was previously observed for folding and binding of p27 to the Cdk2/cyclin A complex [24].

### Mechanism of electrostatically accelerated folding upon encounter

**Figure 5:**
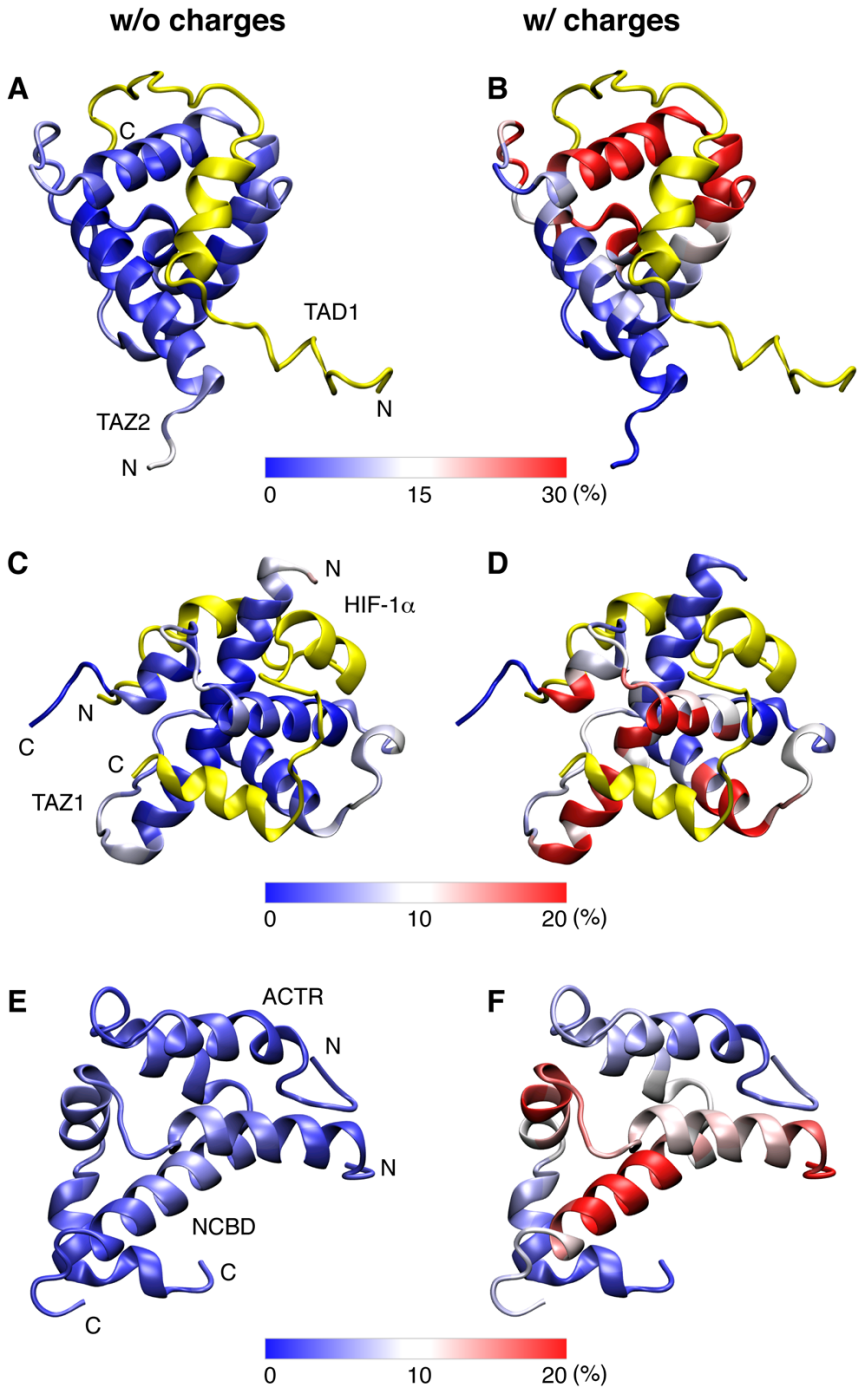
Distributions of IDPs on the substrate surfaces in the collision complexes derived from simulations using models with and without explicit charges. For the p53-TAD1/TAZ2 (A–B) and HIF-1α/TAZ1 (C–D) complexes, TAZ2 and TAZ1 are colored based on the probability of each residue in contact with the IDPs in the collision complex ensembles, and p53-TAD1 and HIF-1α are shown only in the fold and bound conformations (yellow cartoon) for reference. For the NCBD/ACTR complex (E–F), both IDPs are shown in the bound and folded conformations and colored based on the probability of each residue involved (nonspecific) intermolecular contacts in the collision complex ensemble.

**Figure 6:**
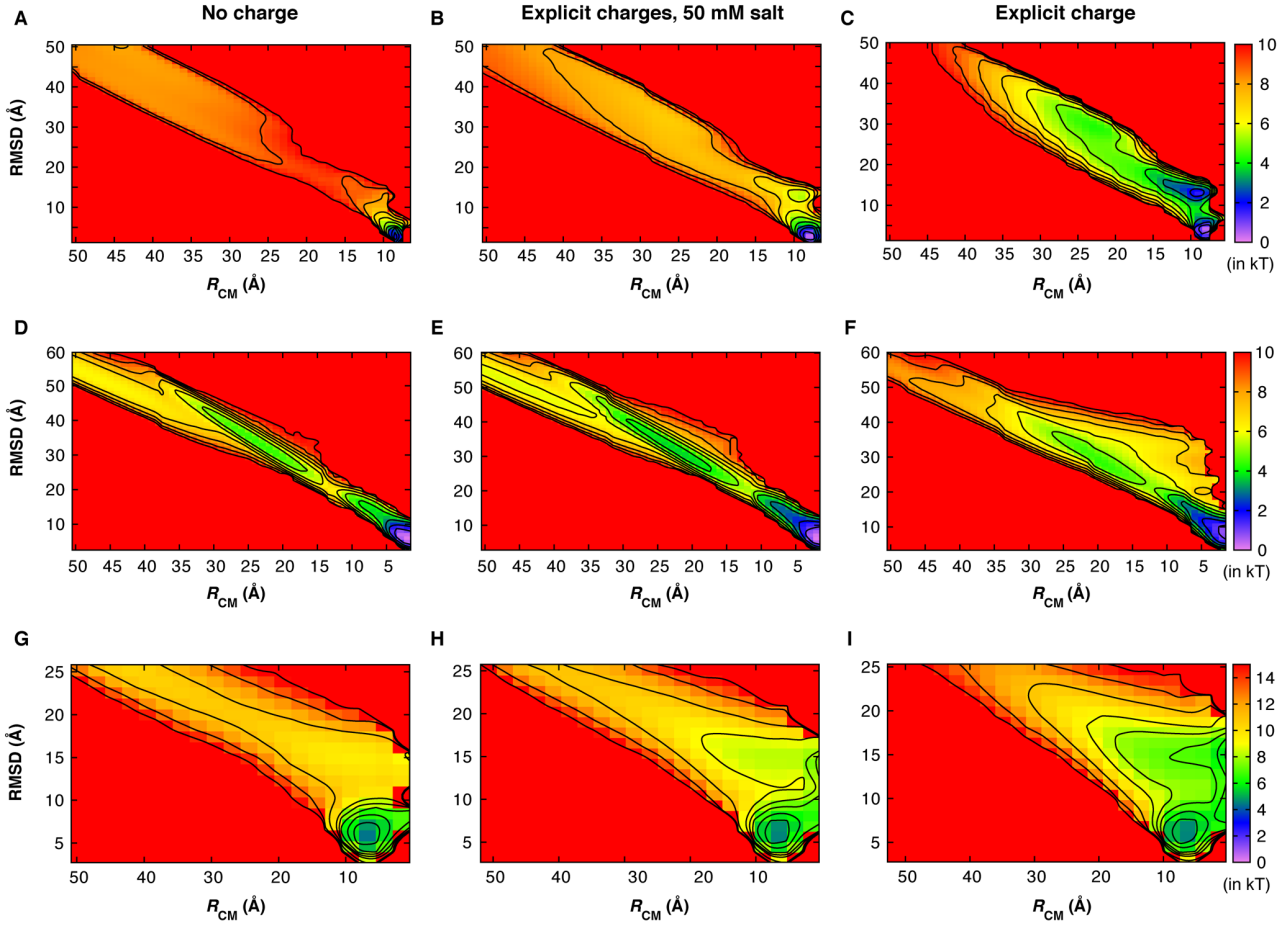
Free-energy surfaces at *T*_m_ as a function of binding RMSD of the IDP and center of mass separation between two peptides (*R*_CM_), computed using various Gō-like models with and without explicit charges and/or 50 mM salt (see Table 2). The binding RMSD (of the IDP) was calculated by first aligning the snapshot with respect to the folded structure using only the folded substrate. For NCBD/ACTR, both proteins are IDPs and the (regular) RMSD was calculated using the whole complex. Rows A–C, D–F and G–I are for the p53-TAD1/TAZ2, HIF-1α/TAZ1 and NCBD/ACTR complexes, respectively. Contours are drawn every kT.

Inspection of the conformational properties of the collision complexes provides further insights into the molecular basis for enhanced efficiency of IDP folding upon encounter due to long-range electrostatic forces. As shown in Fig. 5, without nonspecific electrostatic interactions (models without explicit charges), the initial contacts between two binding partners are largely random, and the distributions of IDP initial contact points on the substrate surface in the collision complexes are relatively uniform (left column). In contrast, with the inclusion of explicit charges, the probabilities of IDP encountering near the native binding interface are dramatically increased. Coupled with reduced escape rates, this allows much higher efficiency of IDP folding upon encounter to achieve higher overall association rate constants (Table 2). The ability of long-range electrostatic forces to guide the recognition process is also reflected in the free energy surfaces as a function of binding RMSD of the IDP and center of mass separation between two peptides. As shown in Fig. 6, long-range electrostatic forces generate a strong free energy gradient that extends over 10–15 Å away from the native bound positions, without creating over-stabilized misfolded states at short separation distances. It is intriguing that, even though both NCBD and ACTR are disordered in the unbound state, nonspecific long-range electrostatic forces between complementary charges on these two proteins can still manage to promote native-like topologies in the collision complexes. In particular, there is a much higher probability of NCBD and ACTR initiating contacts via the C-terminal helix of NCBD and the second helix of ACTR (Fig. 5E–F). This is part of a key pathway of synergistic folding inherent to the NCBD/ACTR complex that was predicted by coarse-grained and atomistic simulations [25], [60] and later substantiated by H/D exchange mass spectrometry [64]. Therefore, nonspecific electrostatic interactions appear to mainly augment existing folding pathways inherent to the folded topologies to facilitate efficient folding of IDPs upon encounter. Coupled with the previous observation that the vicinity of the IDP binding site tends to be enriched with charges to complement those on IDPs [24], thee current results suggest that there is likely a co-evolution of IDP folded topology, charge characteristics, and coupled binding and folding mechanisms. Furthermore, the co-evolution is likely driven by the important need to achieve facile IDP recognition for cellular signaling and regulation.

## Discussion

While fulfilling important functional constraints such as structural plasticity for binding numerous specific targets, protein intrinsic disorder can lead to potential kinetic bottlenecks to be viable in cellular signaling and regulation. Our previous work on the p27/Cdk2/cyclin A complex has revealed a mechanism where nonspecific electrostatic interactions not only enhance the protein-protein encounter kinetics but also promote folding-competent encounter topologies to increase the efficiency of IDP folding upon encounter [24]. Using carefully calibrated topology-based coarse-grained models, we have now further demonstrated that similar electrostatically accelerated encounter and folding mechanisms also underlie the formation of three IDP complexes with more complexed folded structures, namely, p53-TAD1/TAZ2, HIF-1α/TAZ1, and NCBD/ACTR. Importantly, these complexes lack apparent features on the electrostatic surface potentials to directly suggest the ability of nonspecific long-range electrostatic forces to promote native-like encounter topologies to enhance the IDP folding efficiency upon encounter. Nonetheless, there seems to exist a sufficient level of self-consistency between the charge distributions and folded topologies in the bound state to allow accelerated recognition in presence of nonspecific electrostatic interactions. Therefore, enriched charges on IDPs not only play key roles in modulating the conformational properties of the unbound state, but also likely play general and important roles in regulating efficient interactions of IDPs with specific partners. We note that IDPs are frequently regulated by post-translational modifications that add or remove charges. Improved mechanistic understanding of electrostatic forces in IDP recognition derived from the current work will thus help to dissect the profound impacts of post-translational modifications and disease-related mutations on IDP structure and interaction.

## Methods

### Calibration of topology-based coarse-grained models with and without explicit charges

C_α_-only sequence-flavored Gō-like models [67] were first derived from the complex structures of p53-TAD1/TAZ2, HIF1-α/TAZ1 and NCBD/ACTR (see Table 1) using the Multiscale Modeling Tools for Structural Biology (MMTSB) Gō-Model Builder (http://www.mmtsb.org) [68]. The 3 zinc ions bound to TAZ1 in the HIF1-α/TAZ1 complex were modeled explicitly with distance restraints to the coordinating residues. All three models were then calibrated to balance the intrinsic folding propensity and the strength of intermolecular interactions using a previously described protocol [61]. Briefly, the strengths of intra-molecular native contact were uniformly scaled to reproduce the experimentally measured residual helicity of unbound IDPs, which are mainly based on NMR secondary chemical shift and/or circular dichroism analysis (p53-TAD1 [69], NCBD/ACTR [63], and HIF1-α [70]). The residual helicity distributions calculated using the final models listed in Table 2 are provided in Fig. S1. Then, the strengths of intermolecular contacts were adjusted, such that binding affinities calculated from replica exchange molecular dynamics (REX-MD) simulations approximately match the experimental values (see Table 1). Following the previously described procedure [24], the calibrated sequence-flavored Gō-like models were then further modified by assigning proper explicit charges to all charged residues (Lys, Arg, Glu and Asp) as well as zinc ions in the HIF1-α/TAZ1 complex. The charged models were then re-calibrated to reproduce the experimental residual structure level (Fig. S1) and binding affinity (Table 2). Such calibration is critical to avoid inherent bias for particular types of interactions, e.g., intra- vs. inter-molecular or native vs. nonspecific electrostatic. Nonspecific electrostatic interactions were modeled using the Debye-Hückel potential to account for ionic screening. The dielectric constant was set at 80.

### Simulation protocols

The complexes were simulated in cubic boxes with periodic boundary conditions imposed in CHARMM [71], [72]. The box sizes are 100, 100 and 105 Å for p53-TAD1/TAZ2, HIF-1α/TAZ1 and NCBD/ACTR, respectively. Langevin dynamics was performed with 15 fs time steps and a friction coefficient of 0.1 ps^−1^. SHAKE was used to fix all virtual bond lengths [73]. Non-bonded interactions were cut off at 25 Å. Unbound IDPs were simulated at 300 K for 750 ns to calibrate the intramolecular interactions. REX-MD was performed using the MMTSB Toolset [68] for calibration of the intermolecular interactions. For this, eight replicas spanning 270 to 400 K were used. The lengths of REX calibration simulations ranged from 1.05 µs (for p53-TAD1/TAZ2) up to 10 µs (for NCBD/ACTR), as needed for achieving sufficient convergence. Temperature weighted histogram analysis method (WHAM) [74] was used to compute the heat capacity (*C*_V_) curves and generate unbiased probability distributions for free energy and thermodynamic analysis. In particular, the dissociation constants (*K*_D_) were calculated from the bound and unbound probabilities at 300 K [61], where the unbound state was defined as the state without any native intermolecular contacts formed. For NCBD/ACTR complex, the 1D free energy profile lack significant barriers between the unbound and partially bound intermediate states (Fig. 3C, red trace). Therefore, the unbound probability was calculated as 1 – *P*_bound_, where *P*_bound_ is the bound probability (see below for the specific criteria of state assignments). Once calibrated, production simulations of 30–40 µs in lengths were performed using all models at the corresponding *T*_M_'s (see Table 2). The *T*_M_ value was first identified based on the C_V_ curve and then fine tuned to ensure that similar probabilities of sampling the bound and unbound states were observed in the production simulation.

### Free energy and kinetic analysis

All free energy profiles were calculated from the REX simulations and the kinetic analysis was performed based on the production simulations, unless otherwise stated. For calculation of contact fractions, a given native contact was considered as formed if the inter-Cα distance was within 1.0 Å of the distance in the native complex. Nonspecific intermolecular contacts are considered as formed when the inter-Cα distance is within 10 Å cutoff. Three general conformational states were defined for each complex, including the unbound (U), collision complex (CC) and bound (B) states, to understand the effects of electrostatic forces on protein-protein encounter and subsequent folding upon encounter. The unbound state includes conformations with no specific or nonspecific contacts formed between IDP and substrate, and the collision complex state includes conformations with at least one nonspecific but no specific intermolecular contact formed. The bound states are defined as following: 1) for p53-TAD1/TAZ2: *N*_inter_≥11; 2) for HIF-1α/TAZ1: *N*_inter_≥26 for the no charge model, *N*_inter_≥23 for the charged model, and *N*_inter_≥24 for the charged model with 0.05 M salt; 3) for ACTR/NCBD: *N*_inter_≥30. *N*_inter_ is the total number of native intermolecular contacts formed. Note that slightly different criteria were used to define the bound state of HIF-1α/TAZ1 due to small shifts of the bound free energy basins calculated using different models (see Fig. 3). 15-ps running averages were used for assigning states, to avoid including fictitious transitions due to rapid small fluctuations in the calculated contact counts (especially between the U and CC states). The overall on and off rates were calculated directly from the average lifetimes of the bound and unbound states (see Table S4). In addition, MFPTs and numbers of transitions among all three states were derived from the production simulation trajectories, and various rates were calculated as defined in Eqns. 2–4.
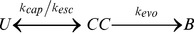
(1)

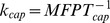
(2)

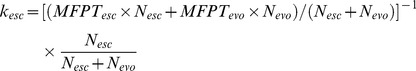
(3)

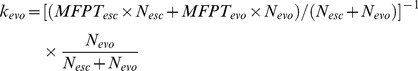
(4)Here, *k*_cap_, *k*_esc_, and *k*_evo_ are the capture, escape (to the unbound state) and evolution (to the bound state) rates of the collision complex, respectively; *N*_esc_ and *N*_evo_ are the numbers of escape and evolution transitions. Note that the MFPTs calculated correspond to the average times spent in an initial state before a transition to the final state. Ideally, the average lifetime of CC should be independent of whether the trajectory ends up in either the U or B state for a true three-state model as shown in Eq. 1. However, the actual transitions between the CC and B states involve several intermediates that are not represented in Eq. 1, and the effective MFPTs as calculated thus depend on both the initial and final states (e.g., see Tables S1, S2, S3). Analytical expressions on similar MFPTs involved in amyloid fibril templating can be found a recent theoretical analysis by Schmit [75]. All molecular visualizations were prepared using VMD [76].

## Supporting Information

Figure S1 Residual helicities of (a) p53-TAD1, (b) HIf-1α, and (c) ACTR in the unbound states calculated using different Gō-like models. The solid traces correspond to models without explicit charges and the dashed traces are from the charged models. The black traces were computed from models with no adjustment of the intramolecular interaction strengths (i.e., scale = 1.0), which significantly over-stabilized the helices. The red traces were calculated using the final calibrated models with optimal scaling of intramolecular interactions (see Table 2 of the main text). The residual helicity showed minimal dependence on the salt concentration for all peptides and the corresponding profiles are thus not shown.
(TIF)

Figure S22D free energy surfaces at *T*_m_ calculated using models with (panels A, C, and E) and without explicit charges (panels B,D, F) (see Table 2 of the main text). 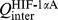 and 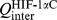 are the fractions of native intermolecular contacts formed by the first and third helices of HIF-1α, respectively. *R*_CM_ is the distance between the centers of mass of HIF-1α and TAZ1. Contours are drawn every kT.(TIF)

Figure S32D free energy surfaces at *T*_m_ calculated using models with (panels A, C, and E) and without explicit charges (panels B,D, F) (see Table 2 of the main text). 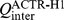,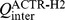 and 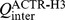 are the fractions of native intermolecular contacts formed by the first, second and third helices of ACTR, respectively. Contours are drawn every kT.(TIF)

Figure S42D free energy surfaces at *T*_m_ calculated using models with (panels A, C, and E) and without explicit charges (panels B,D, F) (see Table 2 of the main text). 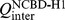,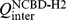 and 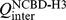 are the fractions of native intermolecular contacts formed by the first, second and third helices of NCBD, respectively. Contours are drawn every kT.(TIF)

Figure S5 Representative snapshots along the binding and folding pathways of p53-TAD1/TAZ2 extracted from the production simulation using the calibration model without explicit charges.
(TIF)

Figure S6 Representative snapshots along the binding and folding pathways for HIF-1α/TAZ1 extracted from the production simulation using the calibration model without explicit charges.
(TIF)

Table S1 MFPTs and numbers of transitions (in parenthesis) between conformational sub-states of the p53-TAD1/TAZ2 complex computed from the production Langevin simulations.
(DOC)

Table S2 MFPTs and numbers of transitions (in parenthesis) between conformational sub-states of the HIF-1α/TAZ1 complex computed from the production Langevin simulations.
(DOCX)

Table S3 MFPTs and numbers of transitions (in parenthesis) between conformational sub-states of the NCBD/ACTR complex computed from the production Langevin simulations.
(DOCX)

Table S4 Averaged on and off rates (*k*_on_ and *k*_off_), as calculated from the mean residence times in either unbound or bound states during the production Langevin simulations at the corresponding *T_m_* (as estimated from short replica exchange simulations).
(DOC)

Text S1 Amino acid sequences of all four IDPs simulated.
(DOC)

## References

[1] Dyson HJ, Wright PE (2005) Intrinsically unstructured proteins and their functions. Nature Reviews Molecular Cell Biology 6: 197–208. 15738986 10.1038/nrm1589

[2] Uversky VN, Oldfield CJ, Dunker AK (2005) Showing your ID: intrinsic disorder as an ID for recognition, regulation and cell signaling. Journal of Molecular Recognition 18: 343–384. 16094605 10.1002/jmr.747

[3] Smock RG, Gierasch LM (2009) Sending signals dynamically. Science 324: 198–203. 19359576 10.1126/science.1169377 PMC2921701

[4] Dunker AK, Lawson JD, Brown CJ, Williams RM, Romero P, et al (2001) Intrinsically disordered protein. Journal of Molecular Graphics & Modelling 19: 26–59. 11381529 10.1016/s1093-3263(00)00138-8

[5] Iakoucheva LM, Brown CJ, Lawson JD, Obradovic Z, Dunker AK (2002) Intrinsic disorder in cell-signaling and cancer-associated proteins. Journal of Molecular Biology 323: 573–584. 12381310 10.1016/s0022-2836(02)00969-5

[6] Uversky VN, Oldfield CJ, Dunker AK (2008) Intrinsically disordered proteins in human diseases: Introducing the D-2 concept. Annual Review of Biophysics 37: 215–246. 10.1146/annurev.biophys.37.032807.125924 18573080

[7] Oldfield CJ, Cheng Y, Cortese MS, Brown CJ, Uversky VN, et al (2005) Comparing and combining predictors of mostly disordered proteins. Biochemistry 44: 1989–2000. 15697224 10.1021/bi047993o

[8] Vacic V, Iakoucheva LM (2012) Disease mutations in disordered regions–exception to the rule? Molecular BioSystems 8: 27–32. 22080206 10.1039/c1mb05251a PMC3307532

[9] Pajkos M, Meszaros B, Simon I, Dosztanyi Z (2012) Is there a biological cost of protein disorder? Analysis of cancer-associated mutations. Molecular BioSystems 8: 296–307. 21918772 10.1039/c1mb05246b

[10] Tsai CJ, Ma B, Sham YY, Kumar S, Nussinov R (2001) Structured disorder and conformational selection. Proteins 44: 418–427. 11484219 10.1002/prot.1107

[11] Tompa P, Szasz C, Buday L (2005) Structural disorder throws new light on moonlighting. Trends in Biochemical Sciences 30: 484–489. 16054818 10.1016/j.tibs.2005.07.008

[12] Boehr DD, Nussinov R, Wright PE (2009) The role of dynamic conformational ensembles in biomolecular recognition. Nat Chem Biol 5: 789–796. 19841628 10.1038/nchembio.232 PMC2916928

[13] Hilser VJ, Thompson EB (2007) Intrinsic disorder as a mechanism to optimize allosteric coupling in proteins. Proceedings of the National Academy of Sciences of the United States of America 104: 8311–8315. 17494761 10.1073/pnas.0700329104 PMC1895946

[14] Shoemaker BA, Portman JJ, Wolynes PG (2000) Speeding molecular recognition by using the folding funnel: The fly-casting mechanism. Proceedings of the National Academy of Sciences of the United States of America 97: 8868–8873. 10908673 10.1073/pnas.160259697 PMC16787

[15] Vacic V, Markwick PRL, Oldfield CJ, Zhao X, Haynes C, et al (2012) Disease-Associated Mutations Disrupt Functionally Important Regions of Intrinsic Protein Disorder. Plos Computational Biology 8: e1002709. 23055912 10.1371/journal.pcbi.1002709 PMC3464192

[16] Chen JH (2012) Towards the physical basis of how intrinsic disorder mediates protein function. Archives of Biochemistry and Biophysics 524: 123–131. 22579883 10.1016/j.abb.2012.04.024

[17] Mittag T, Forman-Kay JD (2007) Atomic-level characterization of disordered protein ensembles. Curr Opin Struct Biol 17: 3–14. 17250999 10.1016/j.sbi.2007.01.009

[18] Galea CA, Wang Y, Sivakolundu SG, Kriwacki RW (2008) Regulation of cell division by intrinsically unstructured proteins: Intrinsic flexibility, modularity, and signaling conduits. Biochemistry 47: 7598–7609. 18627125 10.1021/bi8006803 PMC2580775

[19] Wright PE, Dyson HJ (2009) Linking folding and binding. Current Opinion in Structural Biology 19: 31–38. 19157855 10.1016/j.sbi.2008.12.003 PMC2675572

[20] Click TH, Ganguly D, Chen J (2010) Intrinsically Disordered Proteins in a Physics-Based World. International Journal of Molecular Sciences 11: 5292–5309. 21614208 10.3390/ijms11125292 PMC3100817

[21] Fisher CK, Stultz CM (2011) Constructing ensembles for intrinsically disordered proteins. Current Opinion in Structural Biology 21: 426–431. 21530234 10.1016/j.sbi.2011.04.001 PMC3112268

[22] Das RK, Mittal A, Pappu RV (2013) How is functional specificity achieved through disordered regions of proteins? BioEssays 35: 17–22. 23138868 10.1002/bies.201200115

[23] Zhou HX (2010) From Induced Fit to Conformational Selection: A Continuum of Binding Mechanism Controlled by the Timescale of Conformational Transitions. Biophysical Journal 98: L15–L17. 20303846 10.1016/j.bpj.2009.11.029 PMC2849054

[24] Ganguly D, Otieno S, Waddell B, Iconaru L, Kriwacki RW, et al (2012) Electrostatically Accelerated Coupled Binding and Folding of Intrinsically Disordered Proteins. Journal of Molecular Biology 422: 674–684. 22721951 10.1016/j.jmb.2012.06.019 PMC3432731

[25] Zhang W, Ganguly D, Chen J (2012) Residual structures, conformational fluctuations, and electrostatic interactions in the synergistic folding of two intrinsically disordered proteins. Plos Computational Biology 8: e1002353. 22253588 10.1371/journal.pcbi.1002353 PMC3257294

[26] Kubelka J, Hofrichter J, Eaton WA (2004) The protein folding ‘speed limit’. Current Opinion in Structural Biology 14: 76–88. 15102453 10.1016/j.sbi.2004.01.013

[27] Oldfield CJ, Meng J, Yang JY, Yang MQ, Uversky VN, et al (2008) Flexible nets: disorder and induced fit in the associations of p53 and 14-3-3 with their partners. BMC Genomics 9 Suppl 1: S1. 10.1186/1471-2164-9-S1-S1 PMC2386051 18366598

[28] Huang Y, Liu Z (2009) Kinetic advantage of intrinsically disordered proteins in coupled folding-binding process: a critical assessment of the “fly-casting” mechanism. Journal of Molecular Biology 393: 1143–1159. 19747922 10.1016/j.jmb.2009.09.010

[29] Zhou HX, Pang X, Lu C (2012) Rate constants and mechanisms of intrinsically disordered proteins binding to structured targets. Physical chemistry chemical physics: PCCP 14: 10466–10476. 22744607 10.1039/c2cp41196b PMC3402904

[30] Csermely P, Palotai R, Nussinov R (2010) Induced fit, conformational selection and independent dynamic segments: an extended view of binding events. Trends in Biochemical Sciences 35: 539–546. 20541943 10.1016/j.tibs.2010.04.009 PMC3018770

[31] Brown AM, Zondlo NJ (2012) A Propensity Scale for Type II Polyproline Helices (PPII): Aromatic Amino Acids in Proline-Rich Sequences Strongly Disfavor PPII Due to Proline-Aromatic Interactions. Biochemistry 51: 5041–5051. 22667692 10.1021/bi3002924

[32] Mao AH, Crick SL, Vitalis A, Chicoine CL, Pappu RV (2010) Net charge per residue modulates conformational ensembles of intrinsically disordered proteins. Proceedings of the National Academy of Sciences of the United States of America 107: 8183–8188. 20404210 10.1073/pnas.0911107107 PMC2889596

[33] Muller-Spath S, Soranno A, Hirschfeld V, Hofmann H, Ruegger S, et al (2010) Charge interactions can dominate the dimensions of intrinsically disordered proteins. Proceedings of the National Academy of Sciences of the United States of America 107: 14609–14614. 20639465 10.1073/pnas.1001743107 PMC2930438

[34] Vuzman D, Levy Y (2010) DNA search efficiency is modulated by charge composition and distribution in the intrinsically disordered tail. Proceedings of the National Academy of Sciences of the United States of America 107: 21004–21009. 21078959 10.1073/pnas.1011775107 PMC3000266

[35] Levy Y, Onuchic JN, Wolynes PG (2007) Fly-casting in protein-DNA binding: frustration between protein folding and electrostatics facilitates target recognition. Journal of the American Chemical Society 129: 738–739. 17243791 10.1021/ja065531n

[36] Galea CA, Nourse A, Wang Y, Sivakolundu SG, Heller WT, et al (2008) Role of intrinsic flexibility in signal transduction mediated by the cell cycle regulator, p27(Kip1). Journal of Molecular Biology 376: 827–838. 18177895 10.1016/j.jmb.2007.12.016 PMC2350195

[37] Sivakolundu SG, Nourse A, Moshiach S, Bothner B, Ashley C, et al (2008) Intrinsically unstructured domains of Arf and Hdm2 form bimolecular oligomeric structures in vitro and in vivo. Journal of Molecular Biology 384: 240–254. 18809412 10.1016/j.jmb.2008.09.019 PMC2612038

[38] Arai M, Ferreon JC, Wright PE (2012) Quantitative analysis of multisite protein-ligand interactions by NMR: binding of intrinsically disordered p53 transactivation subdomains with the TAZ2 domain of CBP. Journal of the American Chemical Society 134: 3792–3803. 22280219 10.1021/ja209936u PMC3290704

[39] Rogers JM, Steward A, Clarke J (2013) Folding and Binding of an Intrinsically Disordered Protein: Fast, but Not ‘Diffusion-Limited’. Journal of the American Chemical Society 135: 1415–1422. 23301700 10.1021/ja309527h PMC3776562

[40] Schreiber G, Haran G, Zhou HX (2009) Fundamental Aspects of Protein-Protein Association Kinetics. Chemical Reviews 109: 839–860. 19196002 10.1021/cr800373w PMC2880639

[41] Vijayakumar M, Wong KY, Schreiber G, Fersht AR, Szabo A, et al (1998) Electrostatic enhancement of diffusion-controlled protein-protein association: comparison of theory and experiment on barnase and barstar. Journal of Molecular Biology 278: 1015–1024. 9600858 10.1006/jmbi.1998.1747

[42] Meszaros B, Tompa P, Simon I, Dosztanyi Z (2007) Molecular principles of the interactions of disordered proteins. Journal of Molecular Biology 372: 549–561. 17681540 10.1016/j.jmb.2007.07.004

[43] Liu H, Shi Y, Chen XS, Warshel A (2009) Simulating the electrostatic guidance of the vectorial translocations in hexameric helicases and translocases. Proceedings of the National Academy of Sciences 106: 7449–7454. 10.1073/pnas.0900532106 PMC2678657 19383795

[44] Marcovitz A, Levy Y (2011) Frustration in protein-DNA binding influences conformational switching and target search kinetics. Proceedings of the National Academy of Sciences 108: 17957–17962. 10.1073/pnas.1109594108 PMC3207685 22003125

[45] Azia A, Levy Y (2009) Nonnative electrostatic interactions can modulate protein folding: molecular dynamics with a grain of salt. Journal of Molecular Biology 393: 527–542. 19683007 10.1016/j.jmb.2009.08.010

[46] Stoycheva AD, Onuchic JN, Brooks CL (2003) Effect of gatekeepers on the early folding kinetics of a model beta-barrel protein. Journal of Chemical Physics 119: 5722–5729.

[47] Stoycheva AD, Brooks CL, Onuchic JN (2004) Gatekeepers in the ribosomal protein S6: Thermodynamics, kinetics, and folding pathways revealed by a minimalist protein model. Journal of Molecular Biology 340: 571–585. 15210355 10.1016/j.jmb.2004.04.073

[48] Toth-Petroczy A, Simon I, Fuxreiter M, Levy Y (2009) Disordered tails of homeodomains facilitate DNA recognition by providing a trade-off between folding and specific binding. Journal of the American Chemical Society 131: 15084–15085. 19919153 10.1021/ja9052784

[49] Huang Y, Liu Z (2010) Nonnative interactions in coupled folding and binding processes of intrinsically disordered proteins. PLoS ONE 5: e15375. 21079758 10.1371/journal.pone.0015375 PMC2973977

[50] Chu X, Wang Y, Gan L, Bai Y, Han W, et al (2012) Importance of Electrostatic Interactions in the Association of Intrinsically Disordered Histone Chaperone Chz1 and Histone H2A.Z-H2B. Plos Computational Biology 8: e1002608. 22807669 10.1371/journal.pcbi.1002608 PMC3395605

[51] Kruse JP, Gu W (2009) Modes of p53 Regulation. Cell 137: 609–622. 19450511 10.1016/j.cell.2009.04.050 PMC3737742

[52] Goodman RH, Smolik S (2000) CBP/p300 in cell growth, transformation, and development. Genes & Development 14: 1553–1577. 10887150

[53] Wolynes PG (2005) Recent successes of the energy landscape theory of protein folding and function. Quarterly Reviews of Biophysics 38: 405–410. 16934172 10.1017/S0033583505004075

[54] Dill KA, Chan HS (1997) From Levinthal to pathways to funnels. Nature Structural Biology 4: 10–19. 8989315 10.1038/nsb0197-10

[55] Shea JE, Onuchic JN, Brooks CL (1999) Exploring the origins of topological frustration: Design of a minimally frustrated model of fragment B of protein A. Proceedings of the National Academy of Sciences of the United States of America 96: 12512–12517. 10535953 10.1073/pnas.96.22.12512 PMC22965

[56] Turjanski AG, Gutkind JS, Best RB, Hummer G (2008) Binding-induced folding of a natively unstructured transcription factor. PLoS Comput Biol 4: e1000060. 18404207 10.1371/journal.pcbi.1000060 PMC2289845

[57] Lu Q, Lu HP, Wang J (2007) Exploring the mechanism of flexible biomolecular recognition with single molecule dynamics. Physical Review Letters 98: 128105. 17501161 10.1103/PhysRevLett.98.128105

[58] Wang J, Wang Y, Chu X, Hagen SJ, Han W, et al (2011) Multi-Scaled Explorations of Binding-Induced Folding of Intrinsically Disordered Protein Inhibitor IA3 to its Target Enzyme. Plos Computational Biology 7: e1001118. 21490720 10.1371/journal.pcbi.1001118 PMC3072359

[59] De Sancho D, Best RB (2012) Modulation of an IDP binding mechanism and rates by helix propensity and non-native interactions: association of HIF1alpha with CBP. Molecular BioSystems 8: 256–267. 21892446 10.1039/c1mb05252g

[60] Ganguly D, Zhang W, Chen J (2012) Synergistic folding of two intrinsically disordered proteins: searching for conformational selection. Molecular BioSystems 8: 198–209. 21766125 10.1039/c1mb05156c

[61] Ganguly D, Chen J (2011) Topology-based modeling of intrinsically disordered proteins: balancing intrinsic folding and intermolecular interactions. Proteins 79: 1251–1266. 21268115 10.1002/prot.22960

[62] Cho SS, Levy Y, Wolynes PG (2006) P versus Q: structural reaction coordinates capture protein folding on smooth landscapes. Proceedings of the National Academy of Sciences of the United States of America 103: 586–591. 16407126 10.1073/pnas.0509768103 PMC1334664

[63] Ebert MO, Bae SH, Dyson HJ, Wright PE (2008) NMR relaxation study of the complex formed between CBP and the activation domain of the nuclear hormone receptor coactivator ACTR. Biochemistry 47: 1299–1308. 18177052 10.1021/bi701767j

[64] Keppel TR, Howard BA, Weis DD (2011) Mapping unstructured regions and synergistic folding in intrinsically disordered proteins with amide H/D exchange mass spectrometry. Biochemistry 50: 8722–8732. 21894929 10.1021/bi200875p

[65] Dogan J, Schmidt T, Mu X, Engstrom A, Jemth P (2012) Fast association and slow transitions in the interaction between two intrinsically disordered protein domains. The Journal of biological chemistry 287: 34316–34324. 22915588 10.1074/jbc.M112.399436 PMC3464538

[66] Ubbink M (2009) The courtship of proteins: understanding the encounter complex. FEBS Letters 583: 1060–1066. 19275897 10.1016/j.febslet.2009.02.046

[67] Karanicolas J, Brooks CL (2002) The origins of asymmetry in the folding transition states of protein L and protein G. Protein Science 11: 2351–2361. 12237457 10.1110/ps.0205402 PMC2373711

[68] Feig M, Karanicolas J, Brooks CL (2004) MMTSB Tool Set: enhanced sampling and multiscale modeling methods for applications in structural biology. Journal of Molecular Graphics & Modelling 22: 377–395. 15099834 10.1016/j.jmgm.2003.12.005

[69] Lee H, Mok KH, Muhandiram R, Park KH, Suk JE, et al (2000) Local structural elements in the mostly unstructured transcriptional activation domain of human p53. The Journal of biological chemistry 275: 29426–29432. 10884388 10.1074/jbc.M003107200

[70] Dames SA, Martinez-Yamout M, De Guzman RN, Dyson HJ, Wright PE (2002) Structural basis for Hif-1 alpha/CBP recognition in the cellular hypoxic response. Proc Natl Acad Sci U S A 99: 5271–5276. 11959977 10.1073/pnas.082121399 PMC122759

[71] Brooks BR, Bruccoleri RE, Olafson BD, States DJ, Swaminathan S, et al (1983) Charmm - a Program for Macromolecular Energy, Minimization, and Dynamics Calculations. Journal of Computational Chemistry 4: 187–217.

[72] Brooks BR, Brooks CL, Mackerell AD, Nilsson L, Petrella RJ, et al (2009) CHARMM: The Biomolecular Simulation Program. Journal of Computational Chemistry 30: 1545–1614. 19444816 10.1002/jcc.21287 PMC2810661

[73] Ryckaert JP, Ciccotti G, Berendsen HJC (1977) Numerical-Integration of Cartesian Equations of Motion of a System with Constraints - Molecular-Dynamics of N-Alkanes. Journal of Computational Physics 23: 327–341.

[74] Gallicchio E, Andrec M, Felts AK, Levy RM (2005) Temperature weighted histogram analysis method, replica exchange, and transition paths. Journal of Physical Chemistry B 109: 6722–6731. 10.1021/jp045294f 16851756

[75] Schmit J (2013) Kinetic theory of amyloid fibril templating. Journal of Chemical Physics 138: 185102. 23676074 10.1063/1.4803658

[76] Humphrey W, Dalke A, Schulten K (1996) VMD: Visual molecular dynamics. Journal of Molecular Graphics 14: 33–8, 27–8. 8744570 10.1016/0263-7855(96)00018-5

[77] Feng H, Jenkins LM, Durell SR, Hayashi R, Mazur SJ, et al (2009) Structural basis for p300 Taz2-p53 TAD1 binding and modulation by phosphorylation. Structure 17: 202–210. 19217391 10.1016/j.str.2008.12.009 PMC2705179

[78] Sugase K, Lansing JC, Dyson HJ, Wright PE (2007) Tailoring relaxation dispersion experiments for fast-associating protein complexes. Journal of the American Chemical Society 129: 13406–13407. 17935336 10.1021/ja0762238 PMC2533806

[79] Demarest SJ, Martinez-Yamout M, Chung J, Chen HW, Xu W, et al (2002) Mutual synergistic folding in recruitment of CBP/p300 by p160 nuclear receptor coactivators. Nature 415: 549–553. 11823864 10.1038/415549a

[80] Jo S, Kim T, Iyer VG, Im W (2008) CHARMM-GUI: a web-based graphical user interface for CHARMM. J Comput Chem 29: 1859–1865. 18351591 10.1002/jcc.20945

[81] Jo S, Vargyas M, Vasko-Szedlar J, Roux B, Im W (2008) PBEQ-Solver for online visualization of electrostatic potential of biomolecules. Nucleic Acids Res 36: W270–275. 18508808 10.1093/nar/gkn314 PMC2447802

