# Supplementary figures and images for: Electrostatically Accelerated Encounter and Folding for Facile Recognition of Intrinsically Disordered Proteins
Source: PLoS Comput Biol. 2013 Nov 21;9(11):e1003363. doi: 10.1371/journal.pcbi.1003363 (PMC3836701; doi:10.1371/journal.pcbi.1003363)

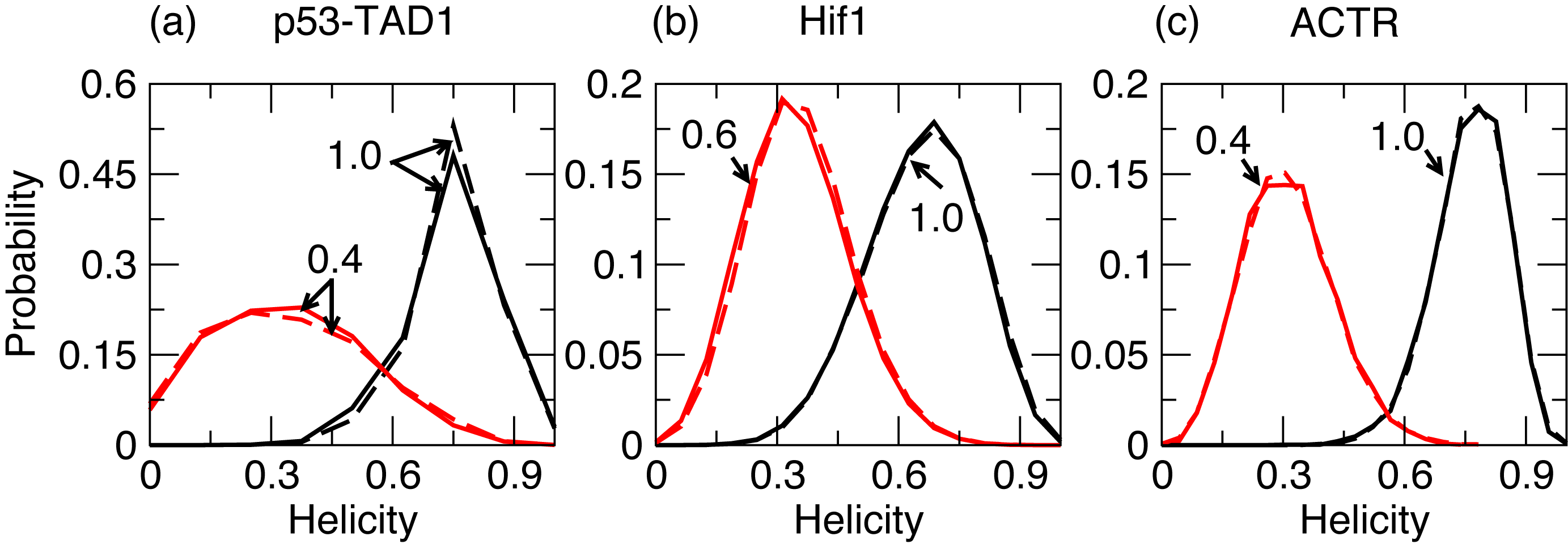

Supplement: Figure S1 — Residual helicities of (a) p53-TAD1, (b) HIf-1α, and (c) ACTR in the unbound states calculated using different Gō-like models. The solid traces correspond to models without explicit charges and the dashed traces are from the charged models. The black traces were computed from models with no adjustment of the intramolecular interaction strengths (i.e., scale = 1.0), which significantly over-stabilized the helices. The red traces were calculated using the final calibrated models with optimal scaling of intramolecular interactions (see Table 2 of the main text). The residual helicity showed minimal dependence on the salt concentration for all peptides and the corresponding profiles are thus not shown. (TIF) [file pcbi.1003363.s001.tif]

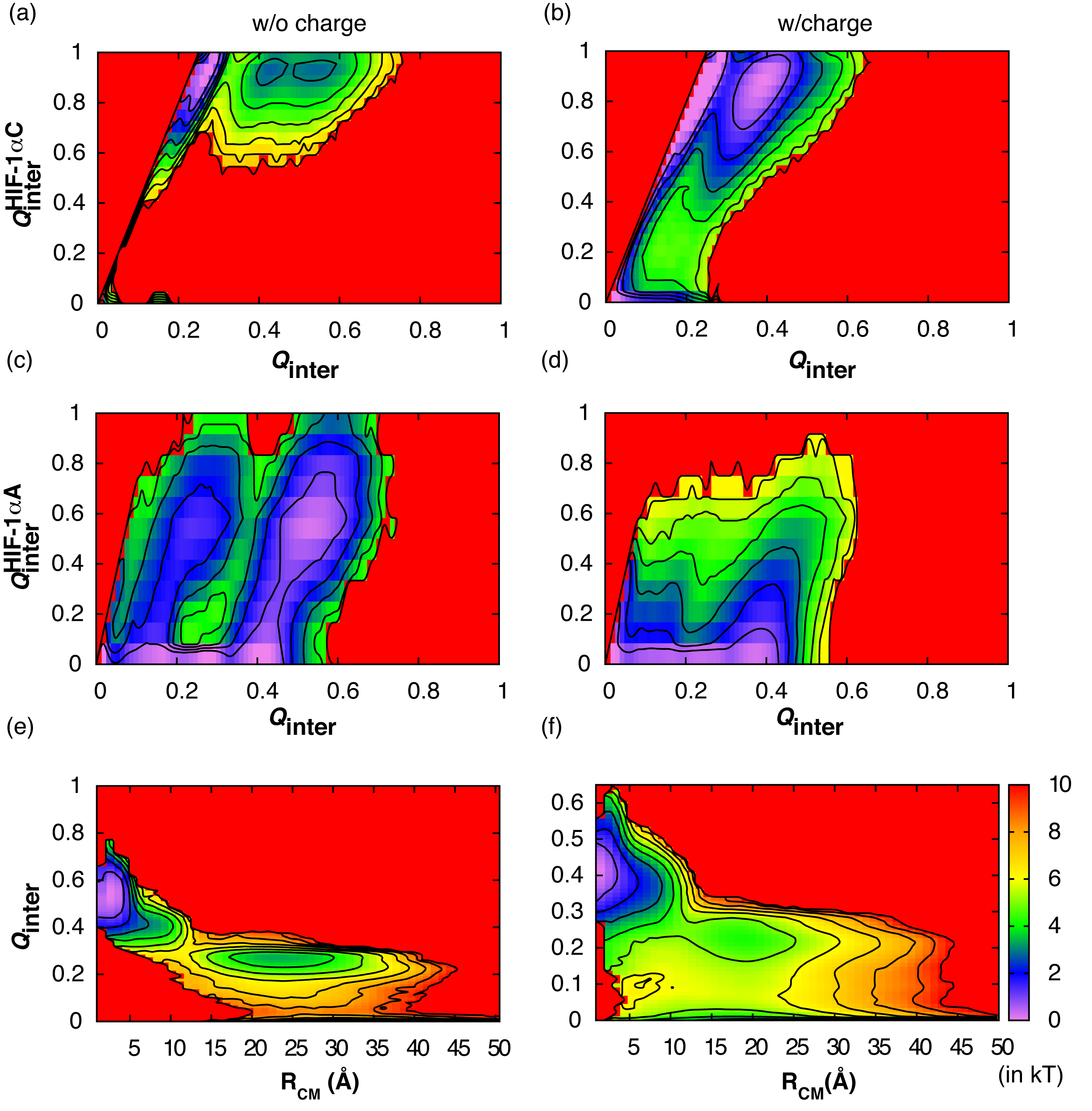

Supplement: Figure S2 — 2D free energy surfaces at T m calculated using models with (panels A, C, and E) and without explicit charges (panels B,D, F) (see Table 2 of the main text). and are the fractions of native intermolecular contacts formed by the first and third helices of HIF-1α, respectively. R CM is the distance between the centers of mass of HIF-1α and TAZ1. Contours are drawn every kT. (TIF) [file pcbi.1003363.s002.tif]

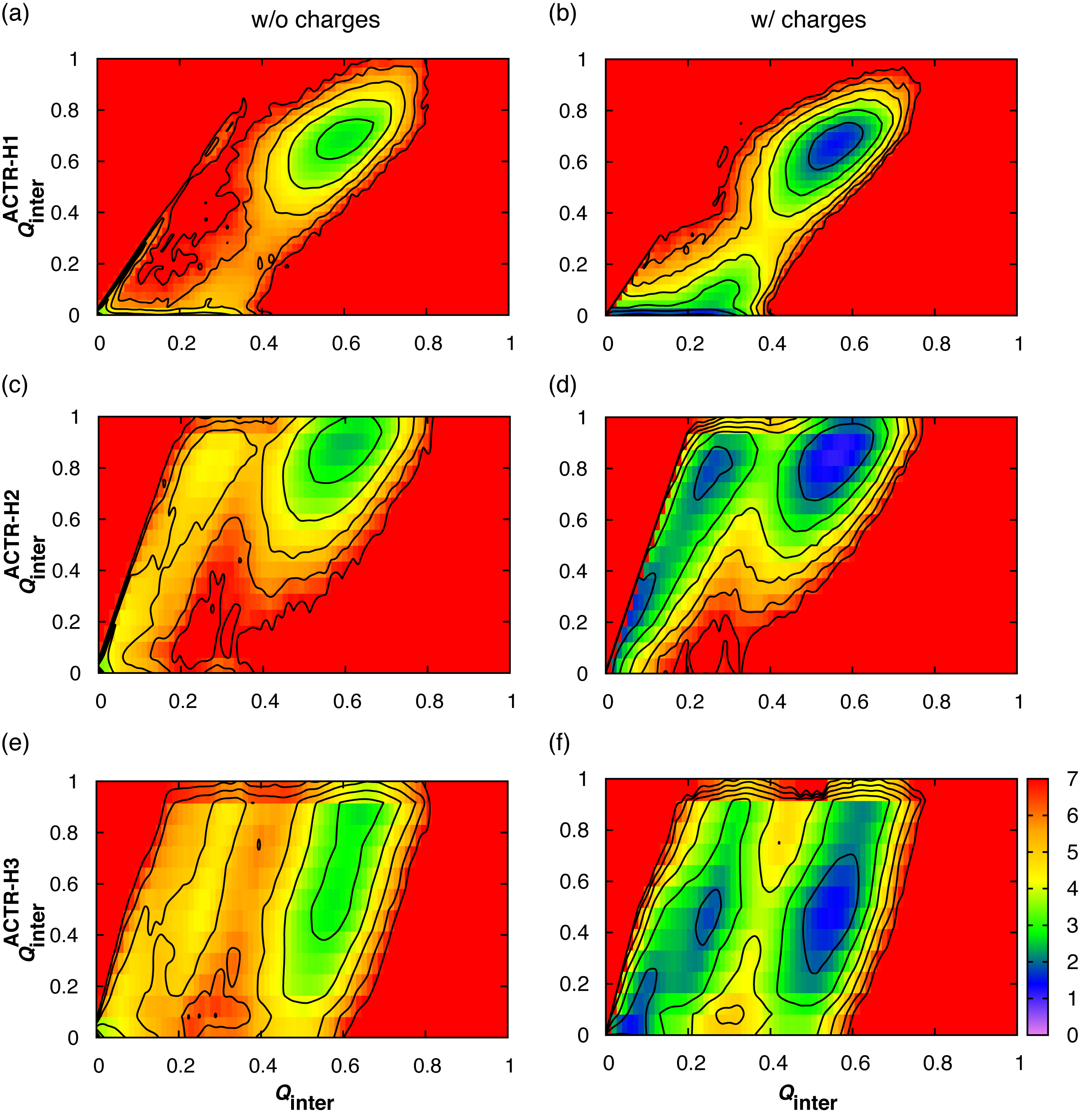

Supplement: Figure S3 — 2D free energy surfaces at T m calculated using models with (panels A, C, and E) and without explicit charges (panels B,D, F) (see Table 2 of the main text). , and are the fractions of native intermolecular contacts formed by the first, second and third helices of ACTR, respectively. Contours are drawn every kT. (TIF) [file pcbi.1003363.s003.tif]

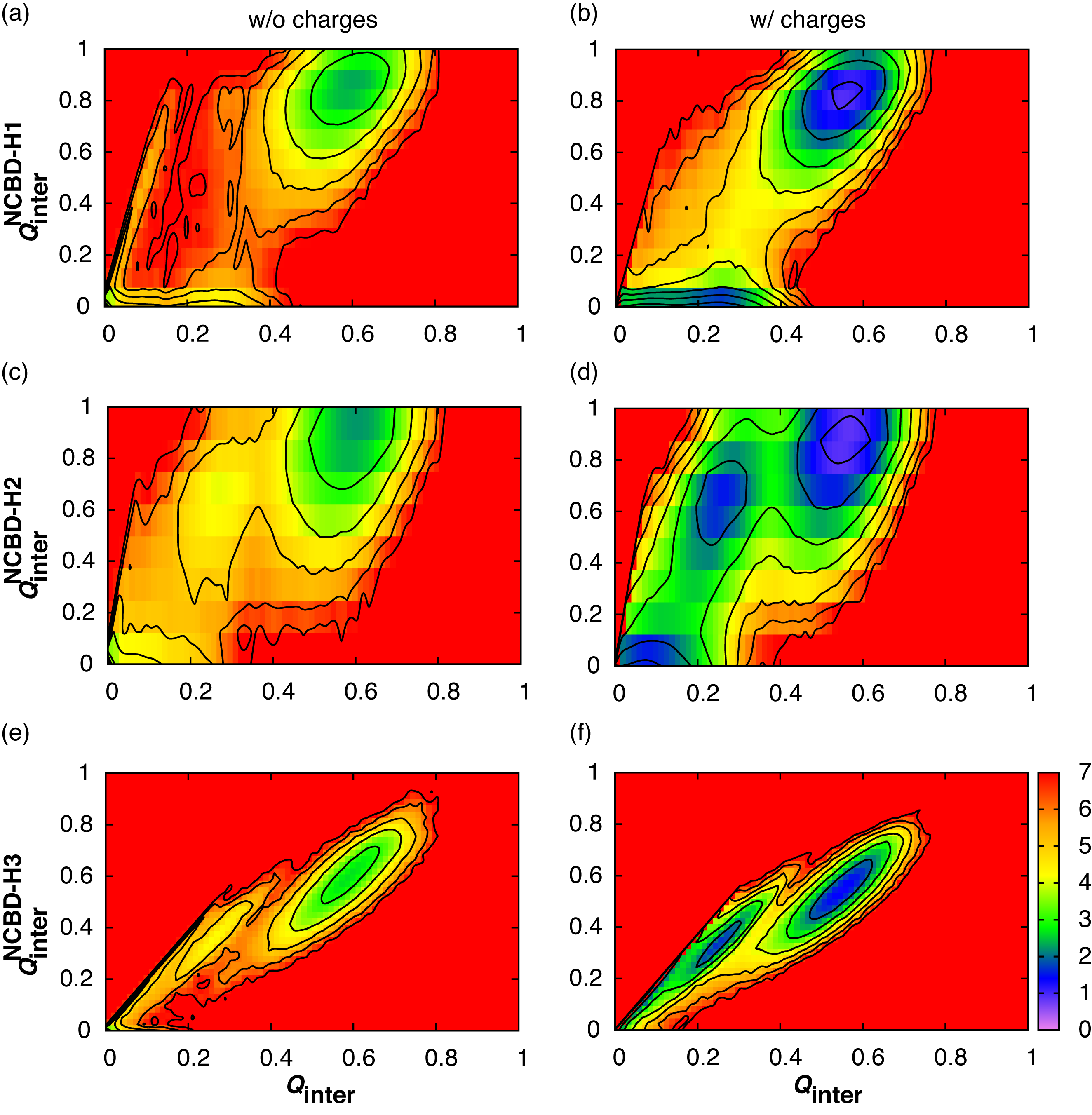

Supplement: Figure S4 — 2D free energy surfaces at T m calculated using models with (panels A, C, and E) and without explicit charges (panels B,D, F) (see Table 2 of the main text). , and are the fractions of native intermolecular contacts formed by the first, second and third helices of NCBD, respectively. Contours are drawn every kT. (TIF) [file pcbi.1003363.s004.tif]

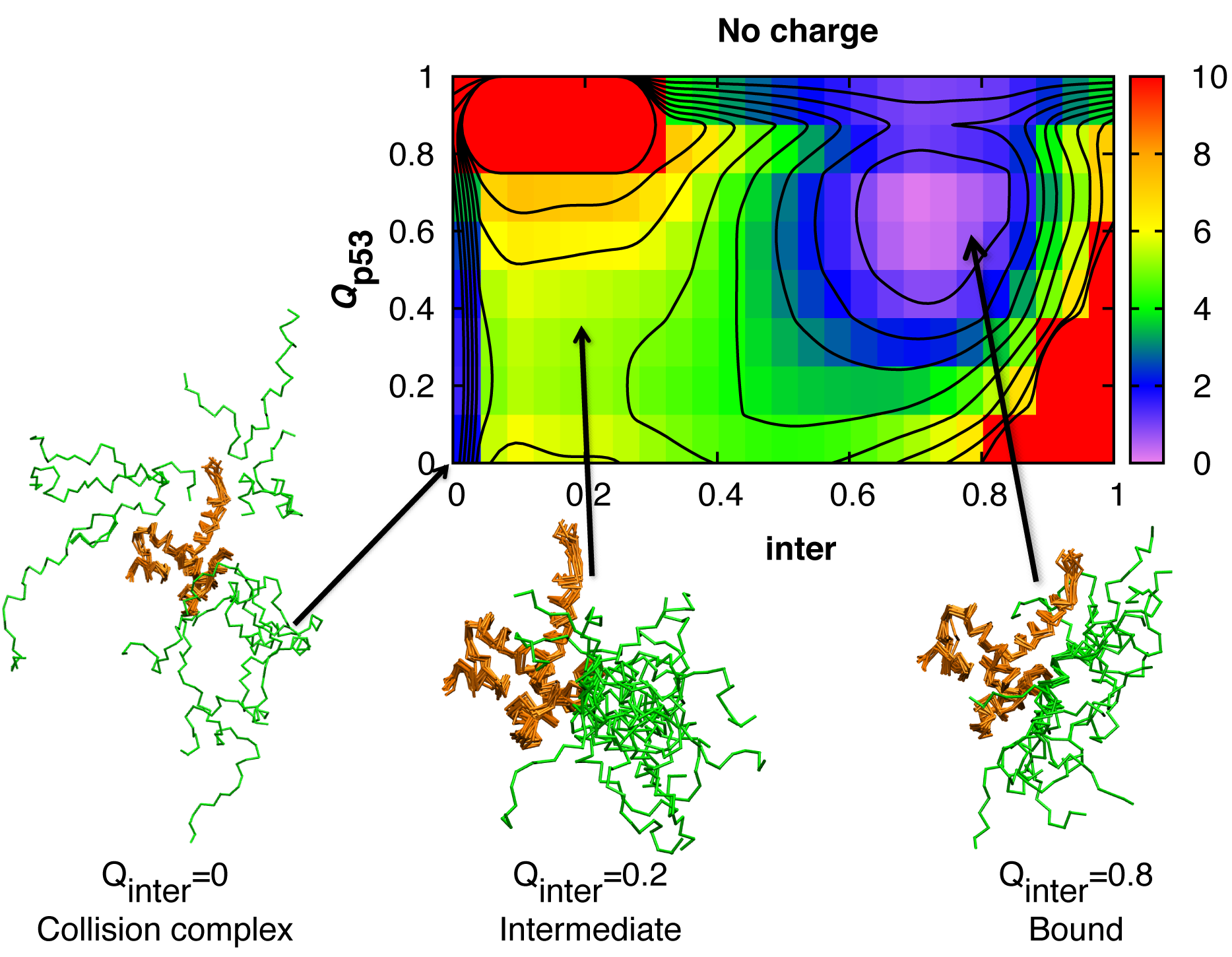

Supplement: Figure S5 — Representative snapshots along the binding and folding pathways of p53-TAD1/TAZ2 extracted from the production simulation using the calibration model without explicit charges. (TIF) [file pcbi.1003363.s005.tif]

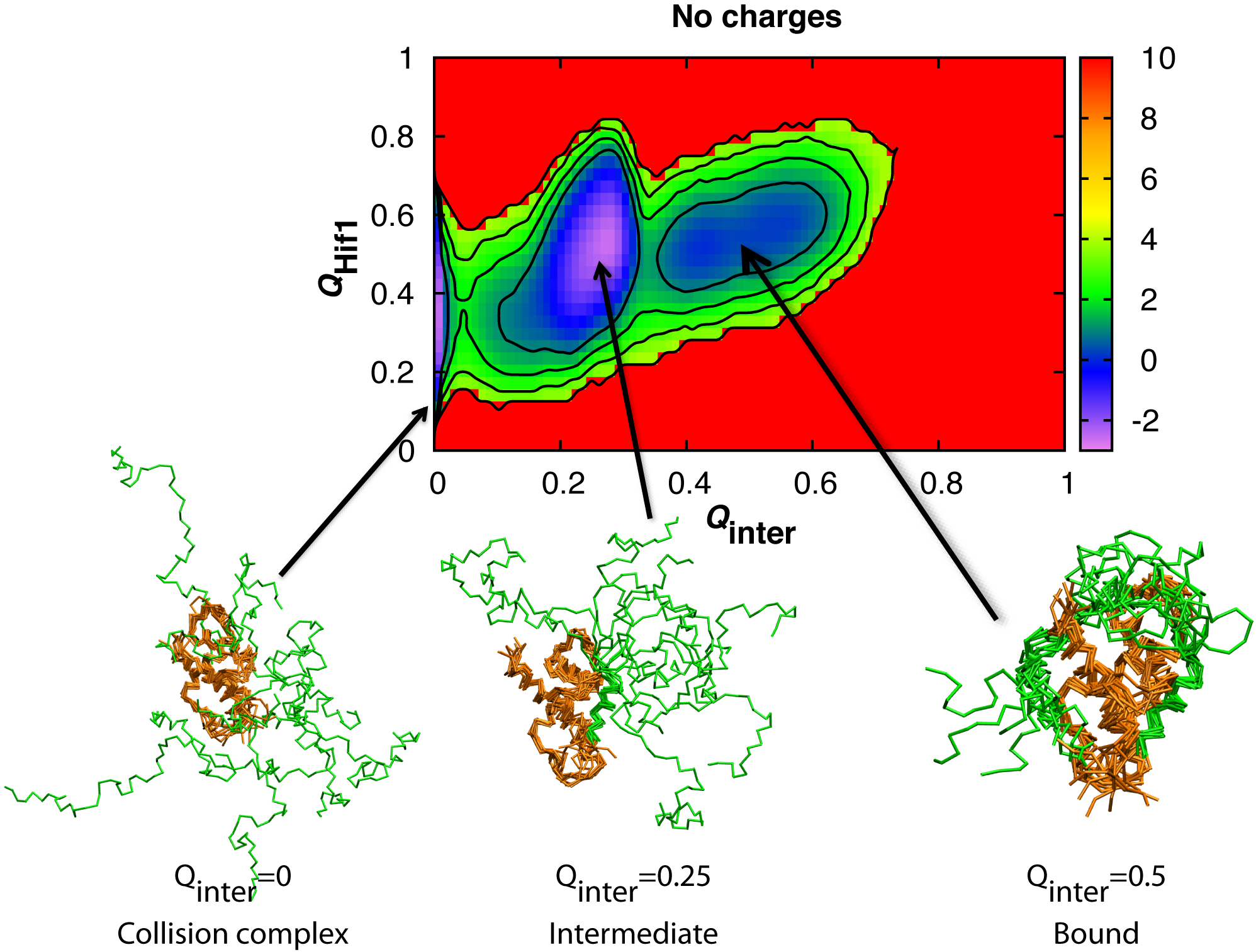

Supplement: Figure S6 — Representative snapshots along the binding and folding pathways for HIF-1α/TAZ1 extracted from the production simulation using the calibration model without explicit charges. (TIF) [file pcbi.1003363.s006.tif]
