# Supplementary material for: Electrostatically Accelerated Encounter and Folding for Facile Recognition of Intrinsically Disordered Proteins
Source: PLoS Comput Biol. 2013 Nov 21;9(11):e1003363. doi: 10.1371/journal.pcbi.1003363 (PMC3836701; doi:10.1371/journal.pcbi.1003363)
Supplement: Table S1 — MFPTs and numbers of transitions (in parenthesis) between conformational sub-states of the p53-TAD1/TAZ2 complex computed from the production Langevin simulations. (DOC) [file pcbi.1003363.s007.doc]

**Table S1**. Mean-first-passage-times (MFPTs) and numbers of transitions (in parenthesis) between conformational sub-states of the p53-TAD1/TAZ2 complex computed from the production Langevin simulations. The state assignment criteria are provided in the main text, and the transitions shown are from the one in the row to that in the column. All MFPTs are in ns.

| **w/o charge**  **w/charge+0.05M salt**  **w/ charge** | **U** | **CC** | **B** |
| --- | --- | --- | --- |
| **U** | - | 0.72 (10026)  0.31 (13735)  0.03 (1098) | - |
| **CC** | 0.12 (10026)  0.24 (13735)  3.47 (1098) | - | 0.26 (60)  0.43 (256)  3.94 (1694) |
| **B** | - | 109.02 (60)  28.86 (256)  2.63 (1695) | - |
