# Supplementary material for: Electrostatically Accelerated Encounter and Folding for Facile Recognition of Intrinsically Disordered Proteins
Source: PLoS Comput Biol. 2013 Nov 21;9(11):e1003363. doi: 10.1371/journal.pcbi.1003363 (PMC3836701; doi:10.1371/journal.pcbi.1003363)
Supplement: Table S4 — Averaged on and off rates (k on and k off), as calculated from the mean residence times in either unbound or bound states during the production Langevin simulations at the corresponding Tm (as estimated from short replica exchange simulations). (DOC) [file pcbi.1003363.s010.doc]

**Table S4**. Averaged on and off rates (*k*_on_ and *k*_off_), as calculated from the mean residence times in either unbound or bound states during the production Langevin simulations at the corresponding *T_m_* (as estimated from short replica exchange simulations). Due to errors in the estimated *T_m_* values as well as limited convergence, the calculated on and off rates are not always similar. Average round trip rates, *k_TS_*, calculated as *N*_TS_/*t*_tot_, are also shown.

| **Models** | ***k*_on_ (μs^-1^)** | ***k*_off_ (μs^-1^)** | $1/\left(k_{on}^{-1}+k_{off}^{-1}\right)$**(μs^-1^)** | ***k*_TS_ (μs^-1^)** |
| --- | --- | --- | --- | --- |
| **TAD1/TAZ2** |  |  |  |  |
| No charge | 6.98 | 9.01 | 3.93 | 4.3 ± 1.5 |
| Charged, 0.05M salt | 32.55 | 30.08 | 15.63 | 14.5 ± 1.1 |
| Explicit charges | 135.77 | 33.08 | 26.60 | 27.0 ± 0.2 |
| **HIF-1α/TAZ1** |  |  |  |  |
| No charge | 6.62 | 14.06 | 4.50 | 6.1 ± 0.5 |
| Charged, 0.05M salt | 14.39 | 21.38 | 8.60 | 10.2 ± 1.8 |
| Explicit charges | 40.9 | 59.8 | 24.29 | 29.4 ± 3.7 |
| **NCBD/ACTR** |  |  |  |  |
| No charge | 1.07 | 1.18 | 0.56 | 0.53 ± 0.2 |
| Charged, 0.05M salt | 3.69 | 3.57 | 1.81 | 1.7 ± 0.1 |
| Explicit charges | 11.02 | 34.5 | 8.35 | 5.2 ± 0.7 |
