# Supplementary material for: Electrostatically Accelerated Encounter and Folding for Facile Recognition of Intrinsically Disordered Proteins
Source: PLoS Comput Biol. 2013 Nov 21;9(11):e1003363. doi: 10.1371/journal.pcbi.1003363 (PMC3836701; doi:10.1371/journal.pcbi.1003363)
Supplement: Text S1 — Amino acid sequences of all four IDPs simulated. (DOC) [file pcbi.1003363.s011.doc]

**Supporting Information**

## **Electrostatically Accelerated Encounter and Folding for Facile Recognition of Intrinsically Disordered Proteins**

Debabani Ganguly, Weihong Zhang and Jianhan Chen*

**1. Sequences of IDPs:** charged residues are highlighted in red/blue fonts, and the folded segments in the complexes are underlined.

**p53-TAD1:**

MET GLU GLU PRO GLN SER ASP PRO SER VAL GLU PRO PRO LEU SER

GLN GLU THR PHE SER ASP LEU TRP LYS LEU LEU PRO GLU ASN ASN

VAL LEU SER PRO LEU PRO SER GLN ALA

**HIF-1α:**

SER ASP LEU ALA CYS ARG LEU LEU GLY GLN SER MET ASP GLU SER

GLY LEU PRO GLN LEU THR SER TYR ASP CYS GLU VAL ASN ALA PRO

ILE GLN GLY SER ARG ASN LEU LEU GLN GLY GLU GLU LEU LEU ARG

ALA LEU ASP GLN VAL ASN

**NCBD:**

PRO ASN ARG SER ILE SER PRO SER ALA LEU GLN ASP LEU LEU ARG

THR LEU LYS SER PRO SER SER PRO GLN GLN GLN GLN GLN VAL LEU

ASN ILE LEU LYS SER ASN PRO GLN LEU MET ALA ALA PHE ILE LYS

GLN ARG THR ALA LYS TYR VAL ALA ASN GLN PRO GLY MET GLN

**ACTR:**

GLU GLY GLN SER ASP GLU ARG ALA LEU LEU ASP GLN LEU HIS THR

LEU LEU SER ASN THR ASP ALA THR GLY LEU GLU GLU ILE ASP ARG

ALA LEU GLY ILE PRO GLU LEU VAL ASN GLN GLY GLN ALA LEU GLU

PRO LYS
